# Extensive Transcriptome Changes During Natural Onset and Release of Vegetative Bud Dormancy in *Populus*

**DOI:** 10.3389/fpls.2015.00989

**Published:** 2015-12-17

**Authors:** Glenn T. Howe, David P. Horvath, Palitha Dharmawardhana, Henry D. Priest, Todd C. Mockler, Steven H. Strauss

**Affiliations:** ^1^Department of Forest Ecosystems and Society, Oregon State UniversityCorvallis, OR, USA; ^2^Biosciences Research Laboratory, United States Department of Agriculture-Agricultural Research ServiceFargo, ND, USA; ^3^Department of Botany and Plant Pathology, Oregon State UniversityCorvallis, OR, USA; ^4^Donald Danforth Plant Science CenterSaint Louis, MO, USA; ^5^Division of Biology and Biomedical Sciences, Washington University in Saint LouisSaint Louis, MO, USA

**Keywords:** chromatin, ecodormancy, endodormancy, gene expression, paradormancy, phytohormone, transcription factor, QTL

## Abstract

To survive winter, many perennial plants become endodormant, a state of suspended growth maintained even in favorable growing environments. To understand vegetative bud endodormancy, we collected paradormant, endodormant, and ecodormant axillary buds from *Populus* trees growing under natural conditions. Of 44,441 *Populus* gene models analyzed using NimbleGen microarrays, we found that 1,362 (3.1%) were differentially expressed among the three dormancy states, and 429 (1.0%) were differentially expressed during only one of the two dormancy transitions (FDR *p*-value < 0.05). Of all differentially expressed genes, 69% were down-regulated from paradormancy to endodormancy, which was expected given the lower metabolic activity associated with endodormancy. Dormancy transitions were accompanied by changes in genes associated with DNA methylation (via RNA-directed DNA methylation) and histone modifications (via Polycomb Repressive Complex 2), confirming and extending knowledge of chromatin modifications as major features of dormancy transitions. Among the chromatin-associated genes, two genes similar to *SPT (SUPPRESSOR OF TY)* were strongly up-regulated during endodormancy. Transcription factor genes and gene sets that were atypically up-regulated during endodormancy include a gene that seems to encode a trihelix transcription factor and genes associated with proteins involved in responses to ethylene, cold, and other abiotic stresses. These latter transcription factors include ETHYLENE INSENSITIVE 3 (EIN3), ETHYLENE-RESPONSIVE ELEMENT BINDING PROTEIN (EBP), ETHYLENE RESPONSE FACTOR (ERF), ZINC FINGER PROTEIN 10 (ZAT10), ZAT12, and WRKY DNA-binding domain proteins. Analyses of phytohormone-associated genes suggest important changes in responses to ethylene, auxin, and brassinosteroids occur during endodormancy. We found weaker evidence for changes in genes associated with salicylic acid and jasmonic acid, and little evidence for important changes in genes associated with gibberellins, abscisic acid, and cytokinin. We identified 315 upstream sequence motifs associated with eight patterns of gene expression, including novel motifs and motifs associated with the circadian clock and responses to photoperiod, cold, dehydration, and ABA. Analogies between flowering and endodormancy suggest important roles for genes similar to *SQUAMOSA-PROMOTER BINDING PROTEIN-LIKE (SPL), DORMANCY ASSOCIATED MADS-BOX (DAM)*, and *SUPPRESSOR OF OVEREXPRESSION OF CONSTANS 1 (SOC1)*.

## Introduction

Dormancy, the temporary suspension of growth (Lang et al., 1987), is a regulated process that controls plant growth, development, and architecture. Lang et al. (1987) subdivided dormancy processes into three types: paradormancy, endodormancy, and ecodormancy. Paradormancy denotes the state in which meristem growth (e.g., in buds or seeds) is inhibited by signals from other plant organs. For example, the shoot apex can inhibit the outgrowth of axillary buds by exerting apical dominance—but this state of paradormancy is released and outgrowth of the axillary buds occurs if the apex is removed. Endodormancy denotes the state in which meristem growth is inhibited by signals within the meristem itself. In plants adapted to cold climates, vegetative buds typically become endodormant in the fall and early winter, and prolonged periods of chilling (i.e., temperatures slightly above freezing) are needed before growth can resume, even under favorable environmental conditions. Even after the release of endodormancy, plants may remain ecodormant because of harsh environmental conditions such as cold or drought that are not conducive to cell division and elongation.

The regulated induction and release of bud endodormancy is critical for the survival and long-term growth of perennial plants in temperate, arid, and semiarid climates. Adaptation to local climatic conditions is generally achieved by natural selection in native populations and by artificial selection in forestry and agricultural populations. However, the matching of plant populations and local climatic cycles may become decoupled with rapid climate change. The induction and release of endodormancy are temporally associated with other changes that confer tolerance to cold and other abiotic stresses. Improved understanding of dormancy-associated gene expression may allow us to manipulate plant populations to speed climatic adaptation, and thus mitigate the adverse effects of climate change on forest and agricultural ecosystems.

Environmental and hormonal signals, including short days (SD), cold, ethylene, gibberellin (GA), and abscisic acid (ABA) play direct roles in growth cessation and bud set (Li et al., 2003; Mølmann et al., 2005; Ruonala et al., 2006). In many trees and other perennial plants, SD and low night temperatures in the fall act synergistically to induce growth cessation, vegetative bud set or shoot-tip abscission, and the first stage of cold acclimation (Howe et al., 1999; Mølmann et al., 2005; Ruttink et al., 2007; Rohde et al., 2011a). In some species (e.g., apple pear, *Populus* sp.), cold temperatures alone can induce growth cessation and endodormancy (Mølmann et al., 2005; Heide, 2008; Rohde et al., 2011a). In the model herbaceous perennial plant, leafy spurge (*Euphorbia esula*), cold night temperatures and long days appear to be most effective for inducing endodormancy (Horvath et al., 2010). Although cold can induce endodormancy in some species, extended chilling temperatures release vegetative bud endodormancy in nearly every temperate perennial species examined (Arora et al., 2003). After the release of endodormancy via chilling, warm temperatures in the spring promote cold deacclimation, vegetative bud flush, and the resumption of elongation growth. The quantitative genetics of bud set and bud flush have been well studied, and many quantitative trait loci (QTL) have been identified (Frewen et al., 2000; reviewed in Howe et al., 2003; Jermstad et al., 2003; Scotti-Saintagne et al., 2004; Pelgas et al., 2011; Rohde et al., 2011b).

The phytochrome photoreceptors and components of the circadian clock regulate short-day-induced dormancy in *Populus* and other perennial plants (Howe et al., 1996; Olsen et al., 1997; Ibanez et al., 2010; Kozarewa et al., 2010). In *Populus*, short-day signals induce growth cessation via a regulatory module consisting of poplar homologs of *CONSTANS (CO)* and *FLOWERING LOCUS T (FT)* in *Arabidopsis* (Bohlenius et al., 2006). Ultimately, SD signals lead to changes in poplar cell proliferation via the *Like-APETALA 1 (LAP1)* gene product, which acts on the AINTEGUMENTA-like 1 transcription factor, which is related to a regulator of cell proliferation in *Arabidopsis* (Azeez et al., 2014). In *Populus*, *FT2* was also induced by chilling, which subsequently led to the induction of 1,3-β-glucanases, reopening of signal conduits, and release of endodormancy (Rinne et al., 2011). The authors hypothesized that the reopened conduits enabled movement of FT2 and CENTRORADIALIS 1 (CENL1) to locations where they promoted bud flush and shoot elongation (Rinne et al., 2011). The expression of other genes that regulate cold acclimation and other endodormancy-associated processes are induced by SD. Transcription factors such as C-REPEAT/DRE BINDING FACTOR 2/DEHYDRATION RESPONSE ELEMENT-BINDING PROTEIN (CBF/DREB) have been implicated in cold acclimation and endodormancy (Doğramaci et al., 2010). For example, overexpression of a *CBF* gene in apple resulted in the ability to induce endodormancy with SDs (Wisniewski et al., 2011).

Many of the same environmental and hormonal signals that regulate dormancy also regulate cold acclimation and flowering. Thus, it is not surprising that the flowering genes *FT2* and *CENL1* also seem to regulate endodormancy (Bohlenius et al., 2006; Ruonala et al., 2008; Hsu et al., 2011; Rinne et al., 2011). Likewise, proteins suspected of regulating *FT2*, such as those encoded by *DORMANCY ASSOCIATED MADS-BOX* (*DAM*) genes, have also been implicated in endodormancy regulation (Bielenberg et al., 2008; Horvath et al., 2010; Sasaki et al., 2011; Yamane et al., 2011). Chromatin remodeling processes associated with vernalization may also regulate bud endodormancy in perennials (Horvath et al., 2003), perhaps by modifying the promoters of *DAM* genes (Horvath et al., 2010; Leida et al., 2012). Indeed, chromatin remodeling seems to accompany changes in *Populus* dormancy states (Vining et al., 2012).

Microarray analysis in *Populus* and several other species have identified common signaling processes associated with endodormancy induction and release (Mazzitelli et al., 2007; Ruttink et al., 2007; Halaly et al., 2008; Horvath et al., 2008; Mathiason et al., 2009; Walton et al., 2009; Campbell et al., 2010; Doğramaci et al., 2010; Karlberg et al., 2010). In addition to flowering genes, genes involved in environmental and phytohormone signaling [e.g., photoperiod, cold, oxidative stress, ethylene, auxin, ABA, and jasmonic acid (JA)], chromatin remodeling, and circadian responses are often differentially expressed during the induction and release of endodormancy. However, only a modest number of genes (<15,000) have been assayed in most previous studies, making it difficult to compare differential expression among gene family members. Furthermore, there are few reports in which endodormancy induction *and* release were compared under natural conditions in the same study.

We used analyses of gene expression to infer physiological processes and *cis*-acting motifs associated with the induction and release of endodormancy in *Populus*. We collected vegetative axillary buds from the end of summer through early spring, and then used a NimbleGen genome-scale microarray to measure global changes in gene expression among dormancy states. Our primary objectives were to: (1) identify which individual genes, biological processes, molecular functions, and regulatory pathways were differentially expressed among dormancy states, (2) classify the differentially expressed genes into contrasting gene expression patterns, and (3) identify *cis*-acting elements associated with each gene expression group. We used this approach and previous observations on dormancy physiology and genomics to better understand the processes regulating endodormancy induction and release in *Populus* trees. We report extensive transcriptome remodeling that both confirm and contradict physiological pathway expectations from the published literature.

## Materials and Methods

### Plant Material

**FIGURE 1:**
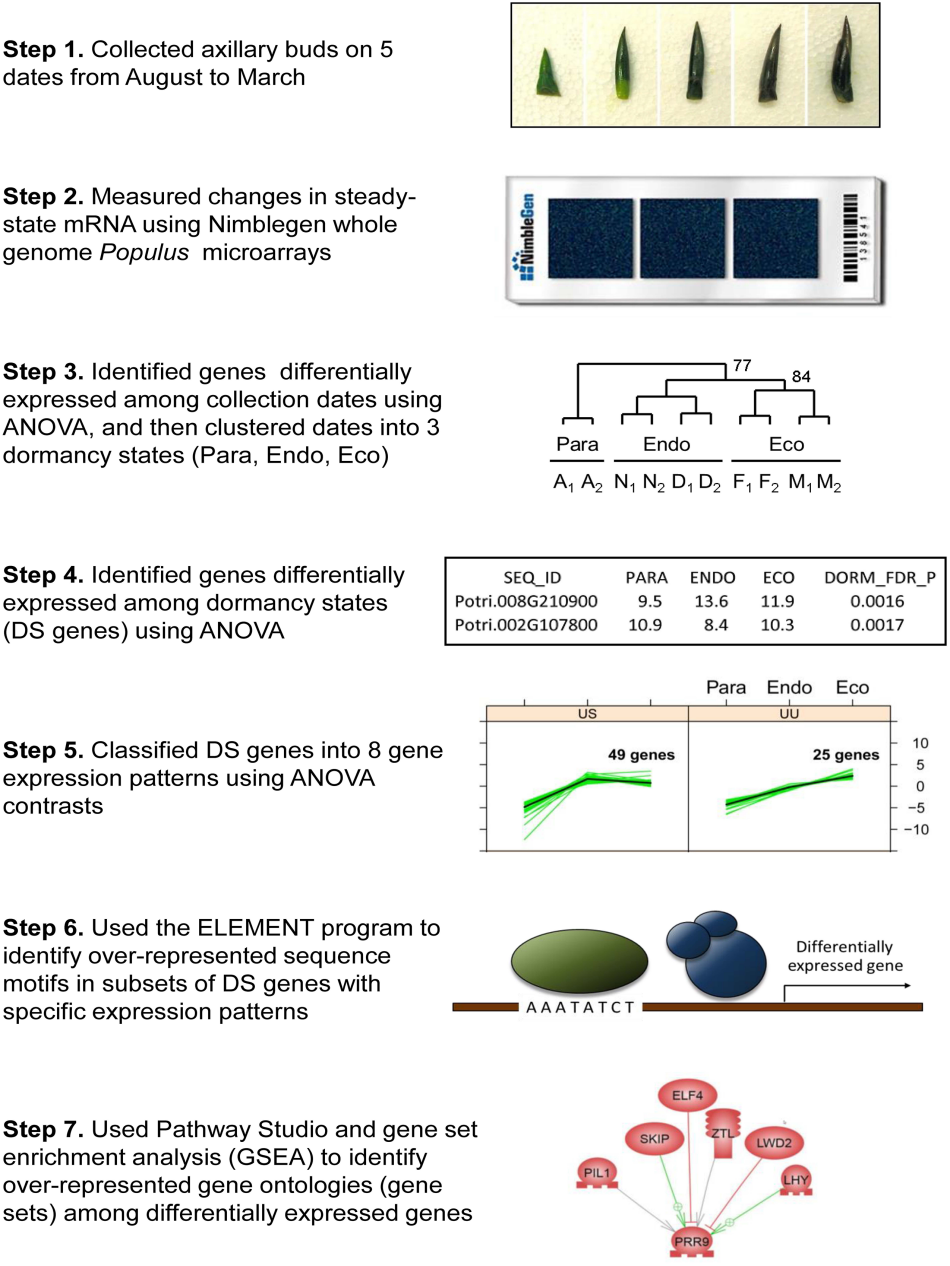
**Flow diagram showing the steps used to analyze dormancy related gene expression in *Populus*.** Step 1 shows representative axillary buds collected in August, November, December, February, and March (left to right). Step 2 shows the NimbleGen gene expression microarray used to measure relative gene expression. Step 3 shows results of clustering five collection time-points into three dormancy states based on the expression of differentially expressed genes. The dormancy states are paradormant (Para), endodormant (Endo), and ecodormant (Eco). Step 4 shows a section of Supplementary Data File 1, which includes results of analyses of variance (ANOVA). Step 5 shows genes that were classified into two of eight gene expression patterns. Step 6 shows a transcription factor binding to an upstream DNA sequence motif (Evening Element). Step 7 shows a representative regulatory network generated by the Pathway Studio program.

We collected axillary buds from the main stem of two, rapidly growing, 3-year-old *Populus trichocarpa* trees (clone Nisqually-1) growing on a field site in Corvallis, OR, USA on five dates between August 2005 and March 2006 (Step 1, **Figure 1**). Average temperatures and precipitation over the collection period are shown in Supplementary Figure S1. Separate RNA isolations were performed on a pooled sample of five buds from each of two trees on each date, resulting in two biological replicates that were used for array hybridizations. The buds were dissected in the field using sterile scalpel blades, immediately frozen in liquid nitrogen, and then subsequently stored at -80°C until they were used for RNA isolation. A few buds collected at the same time were fixed in FAA, dehydrated, and then embedded in wax for sectioning (WAX-IT Histology Services, Vancouver, BC, Canada). De-waxed stem sections were stained with Toluidine Blue-O (Jensen, 1962) and photographed.

### RNA Isolation

RNA was isolated using a Qiagen RNeasy kit according to the manufacturer’s protocol, including a DNase I treatment to remove contaminating genomic DNA (Qiagen, Valencia, CA, USA). The A260/A280 ratios of RNA samples used for hybridizations ranged from 1.8 to 2.0. The absence of contaminating genomic DNA and the integrity of RNA samples were examined by an Agilent 2100 Bioanalyzer (Agilent Technologies, Palo Alto, CA, USA). The RNA Integrity Numbers (RIN; Mueller et al., 2004) of the RNA samples ranged from 8.5 to 10.0, indicating that high-quality RNA was used for the microarray hybridizations.

### Microarray Analysis

We measured gene expression using a microarray designed to target all predicted genes in the *P. trichocarpa* nuclear and organellar genomes plus a set of divergent aspen transcripts (Step 2, **Figure 1**). The microarray, which was manufactured by Roche NimbleGen^1^, was originally designed to target 55,794 nuclear, 59 mitochondrial, 71 chloroplast, and 49 miRNA gene models based on version 1.1 of the *P. trichocarpa* genome sequence (v1.1; Tuskan et al., 2006), plus 9,995 unigenes derived from aspen ESTs (Sterky et al., 2004). With a few exceptions, each original gene model was represented by two copies of three different 60-mer isothermal probes. Original microarray information is archived in the NCBI Gene Expression Omnibus (GEO) database as accession numbers GPL2699 and GPL7424. To analyze the microarray data using the latest gene models, we used BLASTN (Altschul et al., 1990) to reassign the 194,260 *Populus* probe sequences from the original microarray to the transcript sequences from the *Populus* v3.0 genome assembly (file Ptrichocarpa_210_transcript.fa released as part of Phytozome v9.0^2^). We assigned each array probe to one v3.0 gene model, omitting the filtering of low-complexity probes and using a 75% nucleotide identity cutoff. By reassigning the microarray probes to *Populus* v3.0 transcripts, we were able to measure the expression of 35,048 out of 41,335 v3.0 primary transcripts. For probes that could not be assigned to v3.0 transcripts, we retained the original gene model assignment. These 9,393 v1.1 transcripts were also included in the analyses described below.

Biotin-labeled cRNA was produced using the Ambion MessageAmp^TM^ II aRNA amplification kit according to the manufacturer’s instructions (Van Gelder et al., 1990; Life Technologies, 2011), and then sent to NimbleGen for fragmentation, hybridization, and detection. Briefly, total RNA (∼1500 ng) from each sample was reversed transcribed using an oligo(dT) primer with a T7 promoter. After second-strand synthesis, the cDNA was used as a template for synthesizing biotin-labeled antisense RNA (cRNA) using *in vitro* transcription with T7 RNA polymerase. For each sample, ∼20 μg cRNA was sent to NimbleGen for fragmentation, hybridization, and detection as described by Kaushik et al. (2005). After hybridization and washing, the arrays were stained with a streptavidin-Cy3 conjugate, and then scanned with a GenePix 4000B microarray scanner.

The complete microarray dataset was deposited in the GEO database^3^ as accession GPL20616. We created new NimbleGen design files (ndf and ngd files) based on the probe reassignments described above, and then normalized the data using NimbleScan v2.6. Microarray data were log2 transformed, background corrected, and then normalized across all arrays using the Robust Multiple-array Average (RMA; Irizarry et al., 2003).

### Characterization of Bud Dormancy States and Tests of Differential Expression

We assigned a dormancy state to each collection date using ANOVA and cluster analysis in SAS v9.3 (Statistical Analysis System, Cary, NC, USA). First, we used ANOVA and a false discovery rate (FDR) *p*-value < 0.05 to identify genes that were differentially expressed among the collection dates. We then used UPGMA and Neighbor-Joining hierarchical clustering to group the collection dates into dormancy states. The UPGMA analysis clustered the collection dates into three distinct clusters: August, November/December, and February/March, which we refer to as paradormant (Para), endodormant (Endo), and ecodormant (Eco) (Step 3, **Figure 1**; see Results), respectively. In the Neighbor-Joining analysis, the February samples clustered with November and December (rather than with March), but this was only weakly supported (i.e., as compared to the UPGMA alternative). Because the UPGMA assignments were judged to be more biologically accurate (i.e., based on morphological observations and past research on *Populus* dormancy), we used the UPGMA groupings for further analyses. We regrouped the samples based on the UPGMA cluster analysis, and then conducted a second ANOVA on the entire dataset to determine which genes were differentially expressed among the assigned dormancy states (treatments; Step 4, **Figure 1**). All subsequent references to ‘regulated’ or ‘differentially expressed’ genes refer to the set of 1,362 genes that were differentially expressed among dormancy states at an FDR *p*-value < 0.05.

### Gene Expression Patterns and Sequence Motifs

*A priori*, we defined eight potential patterns of gene expression that could occur during two dormancy transitions: Para/Endo and Endo/Eco. For each transition, gene expression may either be up-regulated (U), stay the same (S), or be down-regulated (D), which results in eight possible patterns for two transitions when only the differentially expressed genes are tested (i.e., S/S patterns are not possible). That is, 8 patterns = (3 possible changes for the Para/Endo transition × 3 possible changes for the Endo/Eco transition) – 1 pattern (S/S). For each gene, we determined the *p*-value for each of the eight models using the CONTRAST option of SAS Proc ANOVA, and then used the treatment means and the model *p*-values to assign the gene expression pattern (Step 5, **Figure 1**). Because we were interested in clustering the genes based on directional changes in gene expression (not differences in mean expression), we first normalized the data using the ANOVA mean square error for each gene.

We used the ELEMENT program (Mockler et al., 2007) to identify sequence motifs that were overrepresented in each gene expression group (Step 6, **Figure 1**). These analyses were conducted using 2 kb of upstream sequence relative to the *Populus* v3.0 transcription start site. Motifs were associated with a particular gene expression pattern when the average number of motifs per sequence (MOTIF_MN_HITS) was significant at an FDR *p*-value < 0.05 for only one of the gene expression groups. We inferred potential functions of the motifs using the SIGNALSCAN program and the database of Plant *Cis*-acting Regulatory DNA Elements (PLACE^4^; Higo et al., 1999), and by comparing the motifs to motifs in the PlantCare database^5^) and published literature. We then ranked the motifs based on the number of sequences that contained one or more copies of the motif (SEQ_HIT_P), and identified the top 50 motifs.

### Identification of Key Differentially Expressed Genes

We focused attention on genes encoding transcription factors, and genes associated with chromatin, phytohormone responses, or dormancy-related QTL. For each analysis (subset of genes), we classified the genes into four groups: up- or down-regulated from paradormancy to endodormancy, and up- or down-regulated from endodormancy to ecodormancy. Within each group, we ranked genes by FDR *p*-value, and then focused on the top 15 genes in each group if they had an FDR *p*-value < 0.05.

Chromatin-associated genes were identified using the *Arabidopsis thaliana* chromatin database (ChromDB; Gendler et al., 2008) and by including genes that had “chromatin” or “histone” in the TAIR10 functional annotation (defline) of the *Populus* v3.0 annotation file or in any of the “full name” aliases listed in the TAIR10 gene aliases text file^6^ Transcription factor genes were identified using the *P. trichocarpa* and *A. thaliana* Plant Transcription Factor Databases v3.0 (TFDB; Jin et al., 2014^7^). Because phytohormones are involved in very large signaling networks with substantial cross-talk, we defined phytohormone-associated genes as those having *direct* roles affecting hormone responses via their influence on hormone metabolism (biosynthesis or inactivation), transport, or signaling. This definition encompassed genes that link hormone receptors to primary downstream transcription factors, but excluded genes that regulate hormone metabolic genes, secondary transcription factors, and other downstream response genes. Genes located within dormancy-related QTL were identified by mapping the gene models shown in Supplementary Table S4 of Rohde et al. (2011b) to the *Populus* v3.0 gene models using the gene model aliases shown in Supplementary Data File 1. Some gene models noted by Rohde et al. (2011b) were excluded if they no longer mapped to the same general region of the *Populus* v3.0 genome, and some new v3.0 gene models were added if they were contiguous to the genes previously described by Rohde et al. (2011b). The genes belonging to each of these subsets are identified in Supplementary Data File 1.

### Identification of Differentially Expressed Gene Sets using Gene Set Enrichment Analysis (GSEA)

We used gene set enrichment analysis (GSEA) to identify gene sets that were overrepresented among the differentially expressed genes (Step 7, **Figure 1**). GSEA is a statistical approach for determining whether sets of genes defined a priori (e.g., genes with a common Gene Ontology term) preferentially occur toward the top or bottom of a ranked list of genes (Subramanian et al., 2005). Using the FDR *p*-values, we ranked all genes according to their changes in expression between (1) paradormancy versus endodormancy and (2) endodormancy vs. ecodormancy. For each dormancy transition, we conducted two analyses. First, we analyzed the data considering whether the genes were up-regulated or down-regulated. That is, we subtracted the FDR *p*-value from 1, and then multiplied the result by -1 if the gene was down-regulated. GSEA was then used to identify gene sets that were preferentially located near the top or bottom of this list. Second, we analyzed the data ignoring the direction of change (i.e., no -1 multiplier was used), and then used GSEA to identify genes that were preferentially located near the top of this list. This second analysis was used to identify gene sets whose members contribute to the same biological response via opposite changes in gene expression.

We implemented GSEA using the java application GSEA v2.0.13 (Broad Institute, Cambridge, MA, USA^8^) and 1000 bootstrap replications. We used default parameters, except for setting the minimum and maximum gene set sizes to 5 and 500. Gene sets were considered statistically significant at an FDR *p*-value of 0.10. We analyzed three datasets: two Gene Ontology (GO) datasets (biological process and molecular function GO categories^9^; Gene Ontology Consortium, 2012) and one Pathway Studio (PS) dataset (Pathway Studio v8, Elsevier). For the GO gene sets, *Populus* genes were assigned using the GO terms or *Arabidopsis* gene assignments downloaded from the Phytozome web site (Supplementary Data File 1; Phytozome v9.0^10^). For the Pathway Studio dataset, we combined seven types of pathways. The combined dataset included sets of genes that encode (1) expression targets, (2) miRNA targets, (3) protein modification targets, (4) proteins regulating disease, (5) proteins regulating cell processes, and (6) binding partners and (7) neighbors of key proteins and biological processes.

## Results

### Assignment of Bud Dormancy States

**FIGURE 2:**
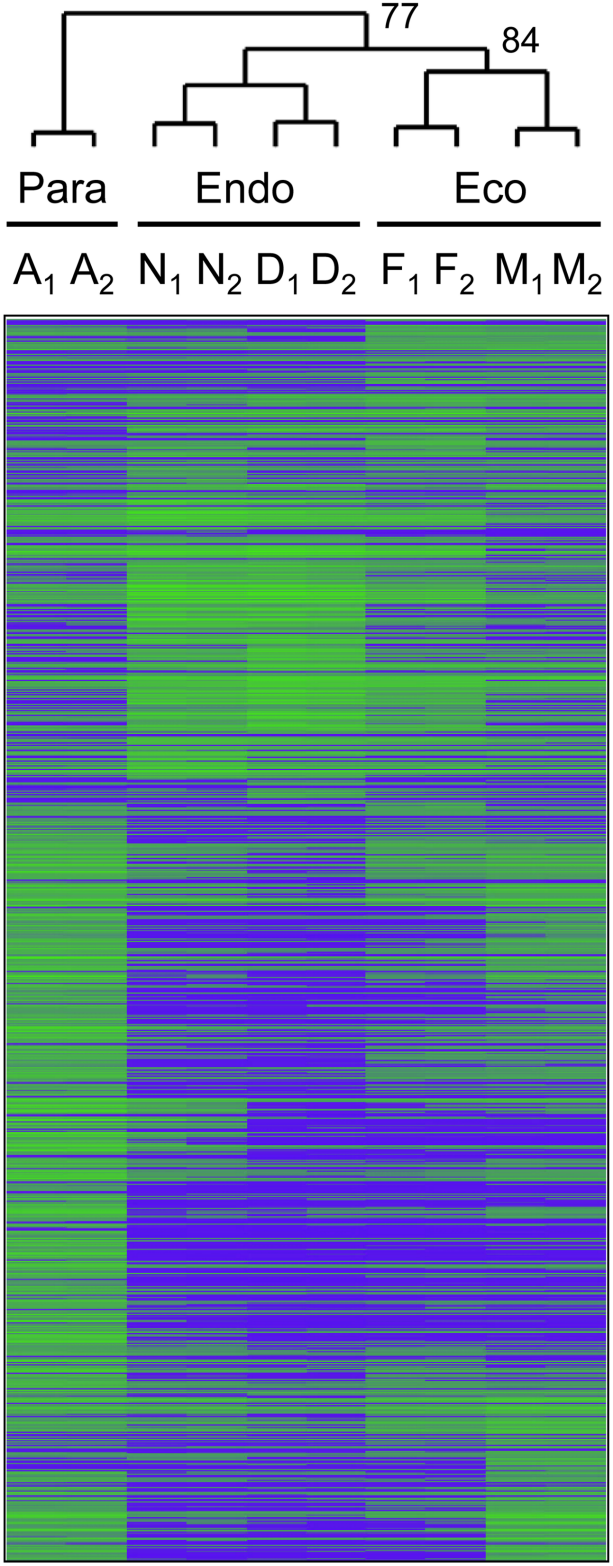
**UPGMA cluster analysis was used to group the 10 samples into three dormancy states: paradormant (Para), endodormant (Endo), and ecodormant (Eco).** Clustering was based on the relative expression of 1,206 genes that were differentially expressed among five collection dates in August (samples A_1_ and A_2_), November (N_1_ and N_2_), December (D_1_ and D_2_), February (F1 and F2), and March (M_1_ and M_2_). Green indicates high relative gene expression and blue indicates low relative gene expression. Bootstrap values (1000 replicates) were 100% for all branch points, except those labeled as 77 and 84%.

We collected axillary buds on five dates between August and March, and then assigned these samples to three dormancy states or treatments based on cluster analysis of gene expression data. Of a total of 44,441 gene models represented on the microarray, 1,206 gene models (36 v1.1 and 1170 v3.0 transcripts) were differentially expressed among months (FDR *p*-value < 0.05; Supplementary Data File 1). Clustering of these differentially expressed genes produced three well-supported groups consisting of samples collected in (1) August, shortly after terminal bud set, (2) November and December, and (3) February and March (**Figure 2**). We classified the first group (August samples) as paradormant (Para), the November and December samples as endodormant (Endo) and the February and March samples as ecodormant (Eco). Morphological observations of bud development (Supplementary Figure S2), and past dormancy research on *P. trichocarpa* and other cottonwoods (see Discussion) support the assignment of these dormancy states.

### Major Transcriptome Changes Occur during Endodormancy Induction and Release

Of the 44,441 gene models analyzed, 1,362 genes (v1.1 = 43; v3.0 = 1,319) were differentially expressed among dormancy states (FDR *p*-value < 0.05; Supplementary Data File 1). Based on analyses of individual dormancy transitions, however, 1,523 genes (v1.1 = 25; v3.0 = 1,498) were differentially expressed between paradormancy and ecodormancy, and the majority of these (*n* = 1,168) were down-regulated. Only 293 transcripts (v1.1 = 13; v3.0 = 280) were differentially expressed between endodormancy and ecodormancy, and the majority of these (*n* = 193) were up-regulated. A total of 190 genes (v1.1 = 4; v3.0 = 186) were differentially expressed during both transitions. Of all the v3.0 differentially expressed genes discussed above, only 3.2% were novel—i.e., had no *Arabidopsis* match (Supplementary Data File 1). We found no evidence that transcripts associated with the mitochondrial, chloroplast, or miRNA gene models were differentially expressed. However, results for the organelle gene models are difficult to interpret because total RNA was reversed transcribed using an oligo(dT) primer.

### Gene Set Enrichment Analysis (GSEA)

In addition to single-gene analyses, we used GSEA to help identify differentially expressed genes sets, which are groups of genes that share a common biological function, chromosomal location, or regulation (Subramanian et al., 2005). Compared to single-gene analyses, GSEA has the potential to identify biologically relevant genes, even when the results of single gene analyses are not statistically significant, and may yield insights that are not obvious from reviewing long lists of statistically significant genes. In particular, GSEA can be valuable for identifying important regulatory pathways. Significant gene sets identified using the GO-term, Pathway Studio, and phytohormone analyses are presented in Supplementary Tables S1–S8, and overrepresented ontologies of particular interest are described below.

### Differential Expression of Chromatin-associated Genes

#### Overview

**FIGURE 3:**
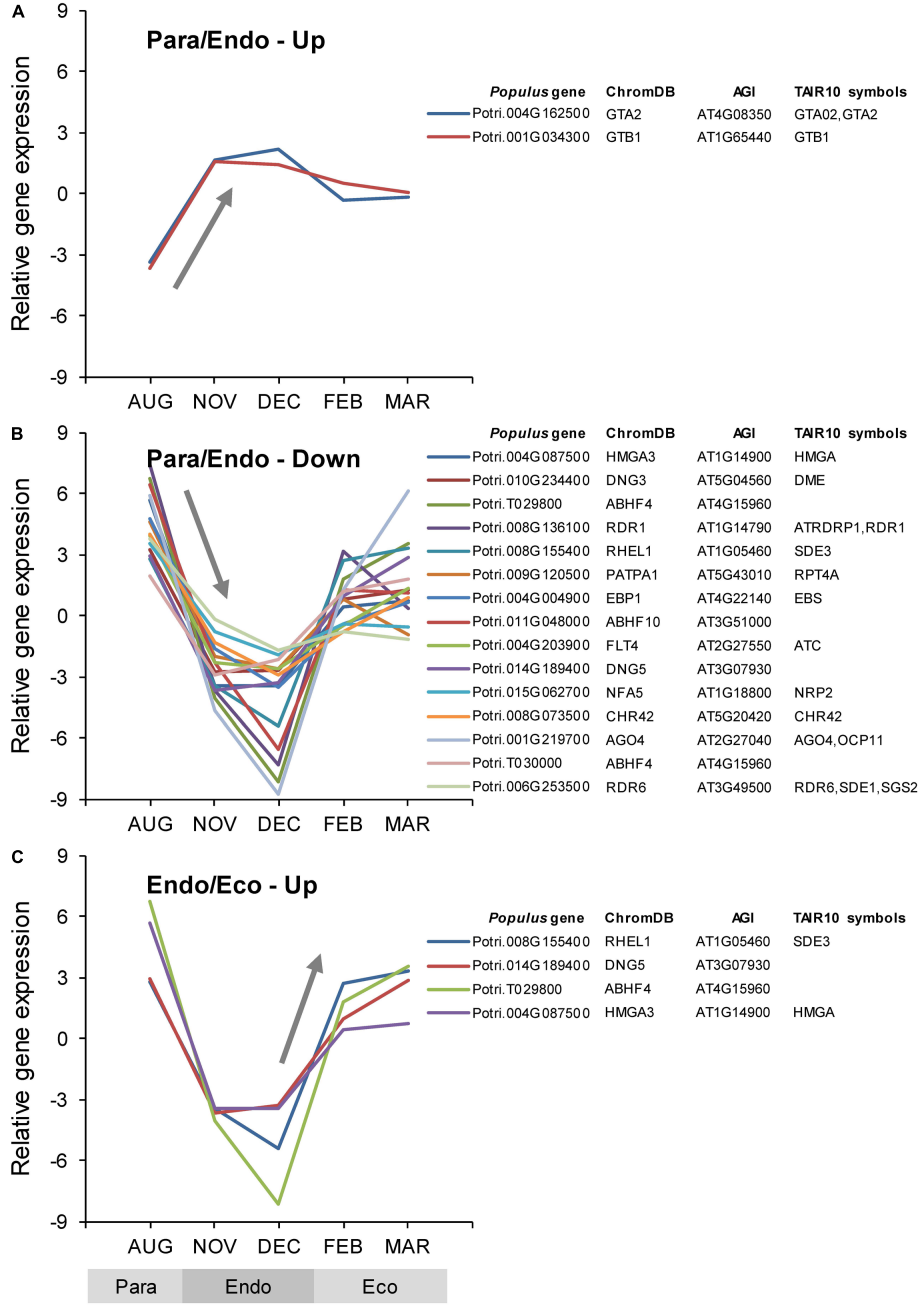
**Relative expression of differentially expressed chromatin-associated genes.** Chromatin-associated genes were identified using the *Arabidopsis thaliana* chromatin database (ChromDB; Gendler et al., 2008). Genes were classified into four groups: up-regulated or down-regulated from paradormancy to endodormancy **(A,B)**, or up-regulated or down-regulated from endodormancy to ecodormancy **(C)**. Within each group, we ranked genes by FDR *p*-value, and then displayed the top 15 genes for each group if they had a FDR *p*-value < 0.05. Only four genes were differentially expressed between endodormancy and ecodormancy, and all of these were up-regulated **(C)**. Gene expression values are the means for each month normalized to a mean of zero and a standard deviation equal to the ANOVA RMSE from the analysis of gene expression differences among months. ChromDB is the ChromDB identifier, *Populus* gene is the *P. trichocarpa* gene-locus name, and AGI and TAIR10 symbols are the *Arabidopsis* gene identifiers and gene symbols from the *P. trichocarpa* v3.0 annotations (http://www.phytozome.net/).

Among the *Populus* v3.0 genes on the array, 727 were identified as being chromatin-associated, 21 of which were differentially expressed between adjacent dormancy states (Supplementary Data File 1). During the transition from paradormancy to endodormancy, 19 genes were down-regulated and 2 were up-regulated. During the transition from endodormancy to ecodormancy, no genes were down-regulated, and 4 were up-regulated. Four genes were differentially expressed during both dormancy transitions. Changes in expression for the top genes (ranked on FDR *p*-value) are shown in **Figure 3**.

#### Chromatin-associated Gene Sets

Three chromatin-associated Pathway Studio gene sets were expressed at lower levels during endodormancy—with significant changes in expression at each of the two dormancy transitions. These gene sets were ‘Neighbors of RDR6’ (RNA-DEPENDENT RNA POLYMERASE 6), ‘Regulators of cytosine methylation,’ and ‘Regulators of maintenance of DNA methylation’ (Supplementary Tables S5 and S6). Sixteen other gene sets were differentially expressed during one of the two dormancy transitions, 13 of which were down-regulated from paradormancy to endodormancy. These Pathway Studio gene sets were ‘Neighbors of SWN’ (SWINGER), ‘Expression targets of RDR6,’ ‘Neighbors of DCL1’ (DICER-LIKE 1), ‘Neighbors of CMT3’ (CHROMOMETHYLASE3), ‘Regulators of DNA methylation,’ ‘Neighbors of HDA6’ (HISTONE DEACETYLASE A6), ‘Neighbors of histone,’ ‘Binding partners of FIE’ (FERTILIZATION INDEPENDENT ENDOSPERM), ‘Expression targets of DET1’ (DEETIOLATED 1), ‘Regulators of chromatin remodeling,’ ‘Neighbors of PRMT11’ (PROTEIN ARGININE METHYLTRANSFERASE 11), ‘Neighbors of AGO7’ (ARGONAUT7), ‘Neighbors of FIE,’ ‘Neighbors of HD1’ (HISTONE DEACETYLASE 1), ‘Neighbors of polycomb complex,’ and the GO molecular function gene set, ‘Histone-lysine *n*-methyltransferase activity.’ Finally, five additional Pathway Studio gene sets were differentially expressed, from paradormancy to endodormancy, but with no common pattern of expression among the gene set members (‘Up- or down-regulated’ gene sets in Supplementary Tables S5 and S6). These gene sets were ‘Binding partners of DDB1A’ (DNA DAMAGE-BINDING PROTEIN 1A), ‘Neighbors of DCL2’ (DICER-LIKE 2), ‘Regulators of histone methylation,’ ‘Binding partners of JAZ10’ (JASMONATE ZIM-DOMAIN PROTEIN 10), and ‘Neighbors of PKL’ (PICKLE).

#### Chromatin-associated Genes

Of the genes shown in **Figure 3**, four genes were down-regulated from paradormancy to endodormancy and then up-regulated from endodormancy to ecodormancy. One of these genes (Potri.004G087500; HMGA3) is similar to a gene that encodes a HIGH MOBILITY GROUP A (HMGA) protein in *Arabidopsis*. The second gene (Potri.008G155400; RHEL1) is similar to *Arabidopsis SILENCING DEFECTIVE 3* (*SDE3*). The third gene (Potri.014G189400; DNG5) is similar to genes that encode DNA glycosylases involved in gene silencing and chromatin remodeling. The last gene (Potri.T029800; ABHF4) is similar to a gene that encodes an alpha/beta-hydrolase in *Arabidopsis*. Two genes had atypical patterns of expression—being strongly up-regulated from paradormancy to endodormancy, and then down-regulated from endodormancy to ecodormancy. The first gene (Potri.004G162500; GTA2), which is listed as encoding a GLOBAL TRANSCRIPTION FACTOR GROUP A2 (GTA2) protein in **Figure 3**, is similar to *Arabidopsis SPT5-2*, an ortholog of yeast *SUPPRESSOR OF TY 5* (Durr et al., 2014). The second gene (Potri.001G034300; GTB1), which is listed as encoding a GLOBAL TRANSCRIPTION FACTOR GROUP B1 (GTB1) protein, is similar to the *SPT6*-like (*SPT6L*) gene from *Arabidopsis* (Li et al., 2010; Gu et al., 2012). Other distinct chromatin-associated genes were also differentially expressed (see ChromDB classifications PATPA1, EPB1, FLT4, NFA5, and CHR42 in **Figure 3**). These genes encode proteins that may be involved in histone ubiquitination and methylation, chromatin assembly or disassembly, histone and DNA binding, and chromatin remodeling.

### Differential Expression of Transcription Factor Genes

#### Overview

**FIGURE 4:**
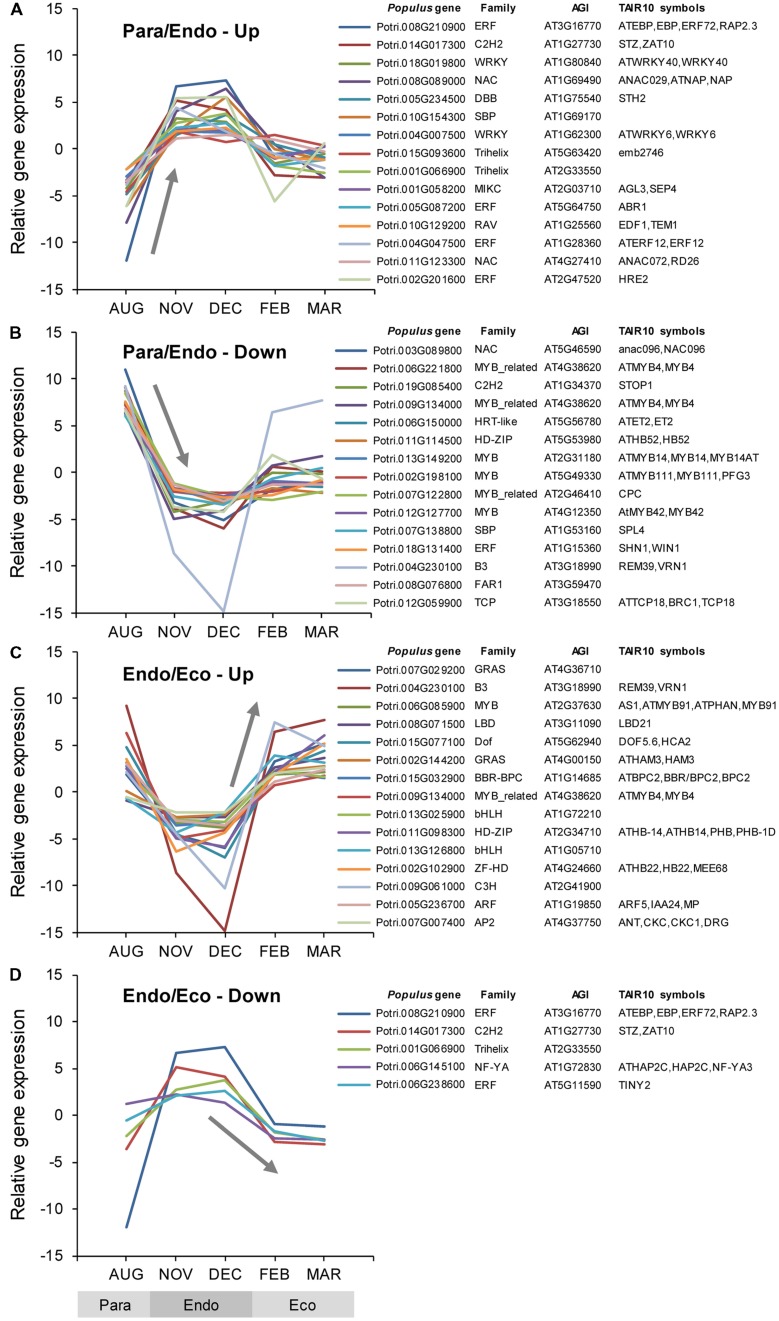
**Relative expression of differentially expressed transcription factor genes.** Transcription factor genes were identified using the *P. trichocarpa* and *A. thaliana* Plant Transcription Factor Databases v3.0 (TFDB; Jin et al., 2014; http://planttfdb.cbi.pku.edu.cn/). Genes were classified into four groups: up-regulated or down-regulated from paradormancy to endodormancy **(A,B)**, or up-regulated or down-regulated from endodormancy to ecodormancy **(C,D)**. Within each group, we ranked genes by FDR *p*-value, and then displayed the top 15 genes for each group if they had a FDR *p*-value < 0.05. Gene expression values are the means for each month normalized to a mean of zero and a standard deviation equal to the ANOVA RMSE from the analysis of gene expression differences among months. Family is the TFDB family designation for the corresponding *Arabidopsis* gene, *Populus* gene is the *P. trichocarpa* gene-locus name, and AGI and TAIR10 symbols are the *Arabidopsis* gene identifiers and gene symbols from the *P. trichocarpa* v3.0 annotations (http://www.phytozome.net/).

Among the *Populus* v3.0 genes on the array, 2,469 were identified as transcription factors, 117 of which were differentially expressed between adjacent dormancy states (Supplementary Data File 1). During the transition from paradormancy to endodormancy, 89 genes were down-regulated and 19 were up-regulated. During the transition from endodormancy to ecodormancy, 5 genes were down-regulated and 19 were up-regulated. Fifteen genes were differentially expressed during both dormancy transitions. Changes in expression for the top genes (ranked on FDR *p*-value) are shown in **Figure 4**.

#### Transcription Factor Gene Sets

Nine transcription factor gene sets were differentially expressed during both dormancy transitions. Four were expressed at higher levels during endodormancy: ‘Neighbors of EIN3’ (ETHYLENE INSENSITIVE 3), ‘Expression targets of EIN3,’ ‘Neighbors of RHL41’ (RESPONSIVE TO HIGH LIGHT 41), and ‘Expression targets of WRKY” (Supplementary Tables S5 and S6). The other five gene sets were expressed at lower levels during endodormancy: ‘Neighbors of JLO’ (JAGGED LATERAL ORGAN), ‘Neighbors of SEU’ (SEUSS), ‘Neighbors of RPL’ (REPLUMLESS), ‘Neighbors of ARF2’ (AUXIN RESPONSE FACTOR 2), and ‘Neighbors of BASIC-HELIX-LOOP-HELIX PROTEIN.’

#### Transcription Factor Genes

Of the genes shown in **Figure 4**, five were differentially expressed during both dormancy transitions. Two genes were down-regulated from paradormancy to endodormancy and then up-regulated from endodormancy to ecodormancy. One of these genes (Potri.009G134000) is similar to a gene that encodes MYB DOMAIN PROTEIN 4 (MYB4) in *Arabidopsis.* The second gene (Potri.004G230100) is similar to *Arabidopsis VERNALIZATION1* (*VRN1*). Three other genes had atypical patterns of expression—being strongly up-regulated from paradormancy to endodormancy, and then down-regulated from endodormancy to ecodormancy. Potri.008G210900 is similar to a gene that encodes an ETHYLENE-RESPONSIVE ELEMENT BINDING PROTEIN (EBP), Potri.014G017300 is similar to the *SALT TOLERANCE ZINC FINGER* (*STZ*) gene, and Potri.001G066900 is similar to an *Arabidopsis* gene that encodes a trihelix transcription factor.

### Differential Expression of Phytohormone-associated Genes

#### Overview

**FIGURE 5:**
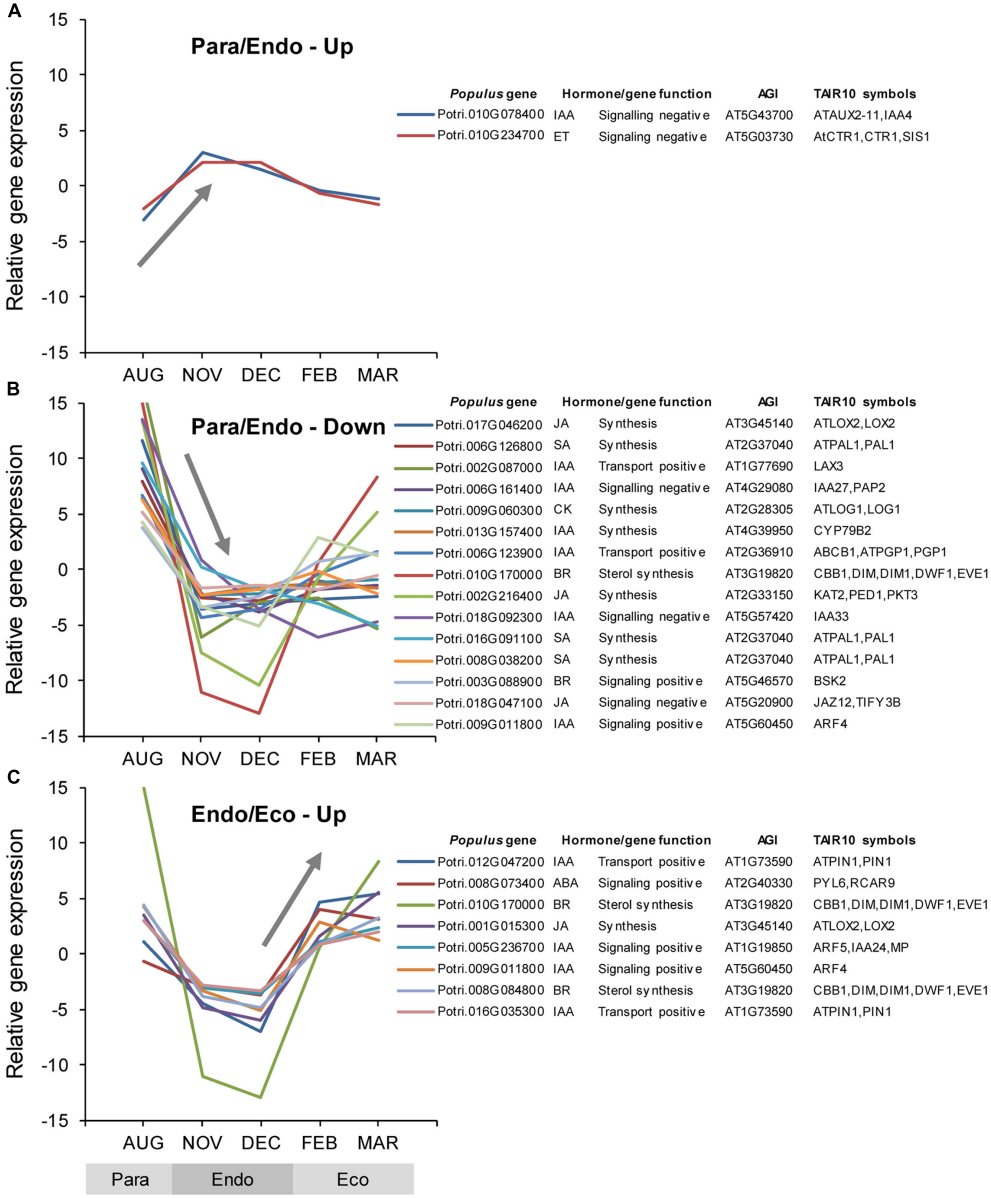
**Relative expression of differentially expressed phytohormone-associated genes.** Genes were classified into four groups: up-regulated or down-regulated from paradormancy to endodormancy **(A,B)**, or up-regulated or down-regulated from endodormancy to ecodormancy **(C)**. Within each group, we ranked genes by FDR *p*-value, and then displayed the top 15 genes for each group if they had a FDR *p*-value < 0.05. All genes differentially expressed between endodormancy and ecodormancy were up-regulated **(C)**. Gene expression values are the means for each month normalized to a mean of zero and a standard deviation equal to the ANOVA RMSE from the analysis of gene expression differences among months. Abbreviations for phytohormones: ABA, abscisic acid; BR, brassinosteroid; CK, cytokinin; ET, ethylene; GA, gibberellin; IAA, indole-3-butyric acid; JA, jasmonic acid, and SA, salicylic acid. *Populus* gene is the *P. trichocarpa* gene-locus name and AGI and TAIR10 symbols are the *Arabidopsis* gene identifiers and gene symbols from the *P. trichocarpa* v3.0 annotations (http://www.phytozome.net/).

Among the *Populus* v3.0 genes on the array, 718 genes were identified as being putatively involved in hormone synthesis, catabolism, signaling, or transport. Of these, 41 were differentially expressed between adjacent dormancy states (Supplementary Data File 1). During the transition from paradormancy to endodormancy, 38 genes were down-regulated and only 2 were up-regulated. No genes were down-regulated from endodormancy to ecodormancy, but 8 genes were up-regulated. Seven genes were differentially expressed during both dormancy transitions. Changes in expression for the top genes (ranked on FDR *p*-value) are shown in **Figure 5**.

#### Auxin-associated Gene Expression

The auxin-associated gene set was mostly down-regulated from paradormancy to endodormancy, and then up-regulated thereafter (Supplementary Tables S7 and S8). Changes in Pathway Studio gene sets provide additional support for the importance of auxin associated genes. One auxin-associated gene set (‘Neighbors of ARF6,’ AUXIN RESPONSE FACTOR 6) was up-regulated from paradormancy to endodormancy, but three other key gene sets, ‘Binding partners of ARF1,’ ‘Neighbors of ARF2,’ and ‘Binding partners of TIR1’ (TRANSPORT INHIBITOR RESPONSE 1), were down-regulated (Supplementary Tables S5 and S6). Two gene sets associated with ARF2 were subsequently up-regulated from endodormancy to ecodormancy.

#### Ethylene-associated Gene Expression

The ethylene gene set was up-regulated from paradormancy to endodormancy, and then down-regulated from endodormancy to ecodormancy (Supplementary Tables S7 and S8). More specifically, one of only two phytohormone genes that were significantly up-regulated from paradormancy to endodormancy is similar to a gene that encodes the CTR1 (CONSTITUTIVE TRIPLE RESPONSE 1) protein, which is a negative regulator of the ethylene response pathway in *Arabidopsis*. Changes in other genes that participate in ethylene responses were described above (see Differential Expression of Transcription Factor Genes).

#### GA-associated Gene Expression

Gibberellin-associated genes were generally up-regulated from paradormancy to endodormancy, but did not change from endodormancy to ecodormancy (Supplementary Tables S7 and S8). We then focused attention on genes encoding GA-20 oxidases and GA-2-oxidases because of their potential involvement in endodormancy. We identified genes encoding GA-20-oxidases and GA-2-oxidases based on similarities to *Arabidopsis* genes and the information presented in Gou et al. (2011), but none was differentially expressed. In fact, no individual GA-related genes were differentially expressed.

#### ABA-associated Gene Expression

The ABA-associated gene set did not change among dormancy states (Supplementary Tables S7 and S8), but our analyses of individual ABA genes identified four genes that were differentially expressed between one or more dormancy states. One gene (Potri.008G0734000), similar to a gene that encodes a PYL (PYRABACTIN RESISTANCE-LIKE) ABA receptor, was down-regulated slightly from paradormancy to endodormancy, and then significantly up-regulated from endodormancy to ecodormancy (**Figure 5**). Three other genes were significantly down-regulated from paradormancy to endodormancy, including genes that likely encode proteins involved in ABA transport (Potri.001G175700), ABA biosynthesis (Potri.003G176300), and positive regulation of ABA signaling (Potri.013G112500). The Pathway Studio gene set, ‘Neighbors of ABF2’ (ABA RESPONSIVE ELEMENTS-BINDING FACTOR 2), was significantly down-regulated from paradormancy to endodormancy. In contrast, the gene set, ‘Neighbors of ABF3,’ was down-regulated from endodormancy to ecodormancy.

#### Brassinosteroid-associated Gene Expression

The brassinosteroid (BR) gene set showed nearly the same expression pattern as the auxin gene set—general down-regulation from paradormancy to endodormancy, and then up-regulation from endodormancy to ecodormancy (but at a FDR *p*-value of 0.115). In addition, the Pathway Studio gene set, ‘Binding partners of BES1’ (BRI1-EMS SUPRESSOR 1), was down-regulated from paradormancy to endodormancy. Analyses of individual genes identified three BR-related genes (Potri.010G170000, Potri.008G084800, and Potri.003G088900) that showed the same general pattern of gene expression (**Figure 5**). The first two genes are similar to *Arabidopsis CABBAGE1 (CBB1)* and the third is similar to *BRASSINOSTEROID-SIGNALING KINASE 2 (BSK2).*

#### Salicylic-acid-associated Gene Expression

Among the phytohormone gene sets, the SA-associated gene set had the strongest evidence for differential expression (Supplementary Tables S7 and S8). Four of the seven SA-associated genes that were differentially expressed were similar to *Arabidopsis PAL1* (*PHENYLALANINE AMMONIA-LYASE 1*), and all of these were down-regulated from paradormancy to endodormancy.

#### Jasmonic-acid-associated Gene Expression

Although the JA-associated gene set was not differentially expressed, the Pathway Studio gene sets, ‘Neighbors of JA,’ ‘Neighbors of MEJA,’ ‘Expression targets of COI1’ (CORONATINE INSENSITIVE 1), and ‘Neighbors of COI1’ were all down-regulated from paradormancy to endodormancy. Furthermore, we saw this same pattern of expression for all six JA-associated genes that were differentially expressed, the top three of which are shown in **Figure 5**. Five of these six genes are associated with JA synthesis (Supplementary Data File 1) and one is associated with negative JA signaling. This latter gene (Potri.018G047100) is similar to *Arabidopsis JASMONATE ZIM-DOMAIN 12 (JAZ12).* Another Pathway Studio gene set, ‘Binding partners of JAZ10,’ was differentially regulated, but with no consistent pattern among the gene set members.

#### Cytokinin-associated Gene Expression

Although the hormone gene set was not differentially expressed, the Pathway Studio gene set, ‘Neighbors of cytokinin,’ and three individual genes (Potri.009G060300, Potri.010G027100, and Potri.016G044100) were all down-regulated from paradormancy to endodormancy.

### Differential Expression of Genes Associated with Bud Set QTL

**FIGURE 6:**
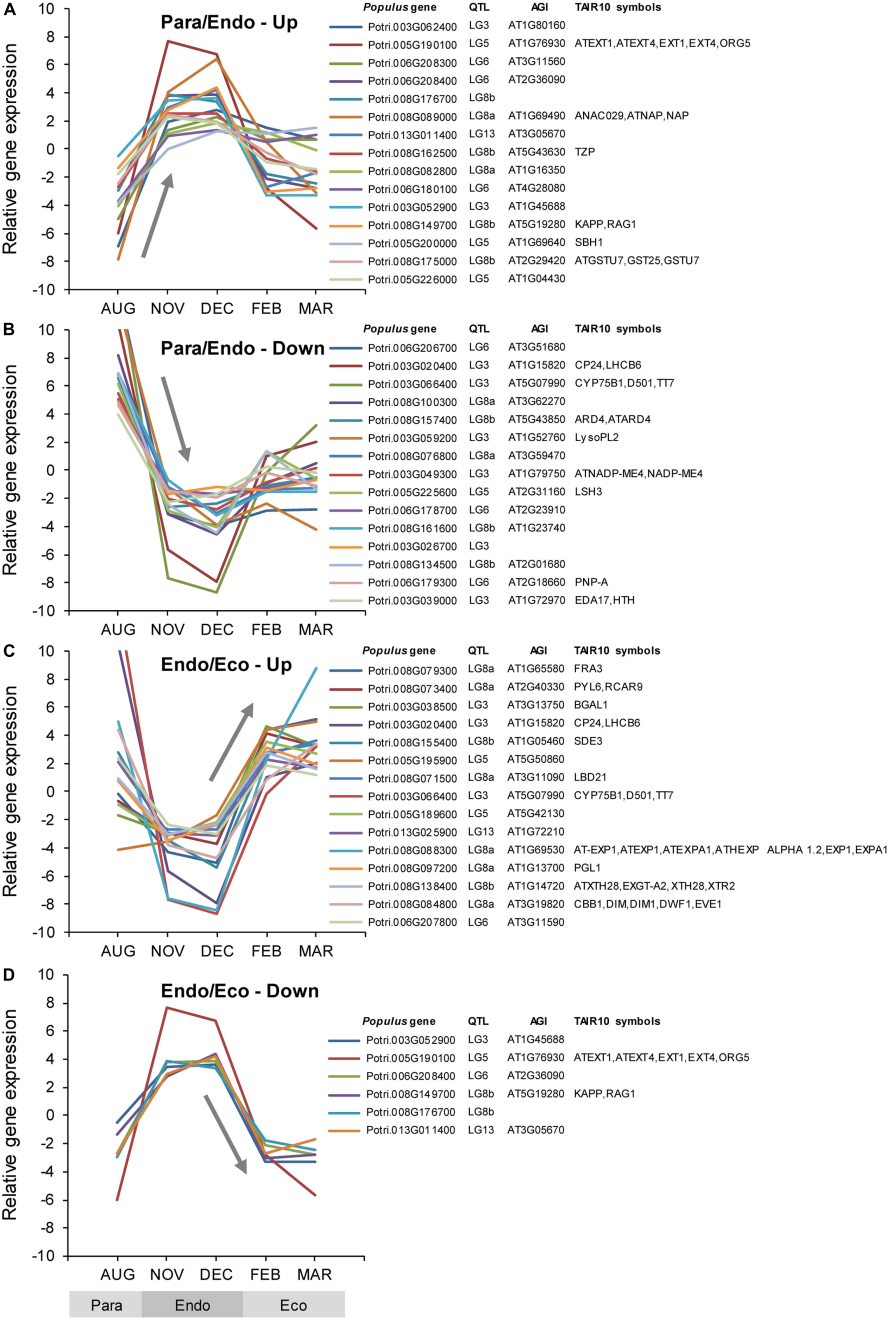
**Relative expression of differentially expressed genes located near dormancy-related QTL.** Genes were classified into four groups: up-regulated or down-regulated from paradormancy to endodormancy **(A,B)**, or up-regulated or down-regulated from endodormancy to ecodormancy **(C,D)**. Within each group, we ranked genes by FDR *p*-value, and then displayed the top 15 genes for each group if they had a FDR *p*-value < 0.05. Gene expression values are the means for each month normalized to a mean of zero and a standard deviation equal to the ANOVA RMSE from the analysis of gene expression differences among months. QTL is the QTL designation from Rohde et al. (2011b), *Populus* gene is the *P. trichocarpa* gene-locus name, and AGI and TAIR10 symbols are the *Arabidopsis* gene identifiers and gene symbols from the *P. trichocarpa* v3.0 annotations (http://www.phytozome.net/).

Among the *Populus* v3.0 genes on the array, 2,181 were identified as being associated with bud set QTL. These genes covered genomic regions ranging from 1.9 Mbp for QTL LG13, to 7.3 Mbp for QTL LG3. A total of 103 genes were differentially expressed using two different criteria. Seventy of these genes were differentially expressed among the three dormancy states (*F*-test), whereas 94 were differentially expressed between either of the two adjacent dormancy states (Supplementary Data File 1). During the transition from paradormancy to endodormancy, 67 genes were down-regulated and 19 were up-regulated. Six genes were down-regulated from endodormancy to ecodormancy, and 15 genes were up-regulated. Fourteen genes were differentially expressed during both dormancy transitions. Changes in expression for the top genes (ranked on FDR *p*-value) are shown in **Figure 6**. Differentially expressed genes were well distributed among the six QTL (described below).

Quantitative trait loci LG3 and LG5 each mapped near 16 differentially expressed genes (i.e., genes differentially expressed among the three dormancy states *or* between adjacent dormancy states), but none had any obvious regulatory role in bud set. In contrast, several potential regulatory genes were found among the 19 differentially expressed genes located near QTL LG6. These include genes that seem to encode a chromatin-associated DCL protein (Potri.006G188800) and a JAZ protein involved in JA signaling (Potri.006G217200; discussed above). Among the 19 differentially expressed genes located near QTL LG8a, are genes that seem to encode proteins involved in chromatin remodeling (Potri.008G073500), positive ABA signaling (Potri.008G073400), BR synthesis (Potri.008G084800), responses to far-red light (Potri.008G076800), organization of lateral organ boundaries (Potri.008G071500), and a NAC-domain transcription factor associated with leaf senescence (Potri.008G089000). QTL LG8b maps near 24 differentially expressed genes, including two chromatin-associated genes (Potri.008G155400 and Potri.008G136100), an Aux/IAA gene (Potri.008G172400), and a gene similar to *MITOTIC ARREST-DEFICIENT 2* (Potri.008G179600). Nine differentially expressed genes mapped near QTL LG13, including a gene (Potri.013G011400) that seems to encode a plant homeodomain (PHD) finger family protein that was up-regulated during endodormancy, and a gene (Potri.013G025900) that may encode a bHLH transcription factor that was strongly down-regulated and mapped near the center of the QTL.

### Patterns of Gene Expression and Regulatory Motifs

**FIGURE 7:**
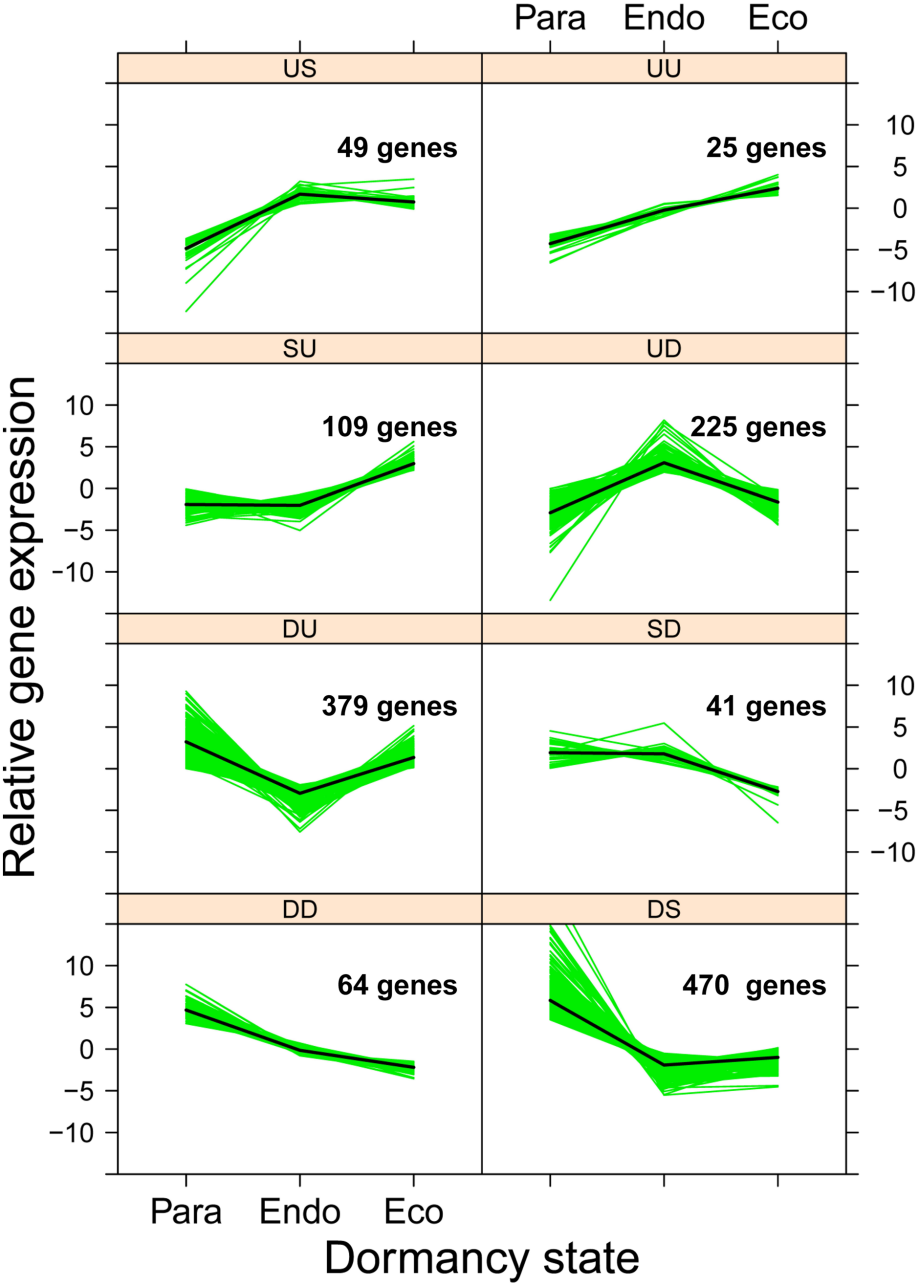
**Expression patterns of 1,362 genes that were differentially expressed among dormancy states.** Genes were classified into eight gene expression patterns associated with two dormancy transitions: paradormancy to endodormancy and endodormancy to ecodormancy. For each transition, gene expression was either up-regulated (U), the same (S), or down-regulated (D). Solid green lines represent the mean normalized expression for each gene, and the solid black lines represent the mean expression of all genes shown in the panel (i.e., gene expression group).

With three dormancy states, eight different patterns of gene expression are possible (**Figure 7**). We used ANOVA to identify genes that were differentially expressed among dormancy states (FDR *p*-value < 0.05), and then used contrast statements to assign each gene to a specific pattern of gene expression. The numbers of genes assigned to each expression pattern ranged from 25 to 470 (**Figure 7**); the gene expression pattern assigned to each gene is available in Supplementary Data File 1.

We subsequently tested whether genes sharing a common pattern of gene expression have common upstream regulatory motifs. For each gene expression group, we analyzed 2 kb of upstream sequence, identifying 315 unique, overrepresented sequence motifs (Supplementary Data File 2). We found only a few unique motifs for four expression patterns (SU and US = 2; SD and UU = 3). Larger numbers of motifs were found for two expression patterns (DD = 16 and DU = 64). The largest numbers of motifs were found for patterns UD (*n* = 103) and DS (*n* = 122). The putative functions of the top 50 overrepresented motifs are shown in **Table 1**.

**Table 1:** Top 50 upstream sequence motifs overrepresented in eight gene expression pattern-groups.

Motif no.	Pattern	Sequence motif	Seq *p*-value	Motif *p*-value	Place sites and other motifs	Proposed functions
208	UD	AAATATCT	5.82E-09	1.63E-14	GATABOX, ROOTMOTIFTAPOX1, EVENINGELEMENTLIKE^∗^	Cold, light, and circadian responses
214	UD	GCCGAC	1.51E-08	4.92E-08	LTRECOREATCOR15, DRECRTCOREAT, CBFHV	Cold, dehydration responses
143	DU	AAAAATCA	2.26E-08	1.50E-07	ARR1AT	Cytokinin response
211	UD	ACGTGTCC	7.87E-08	1.59E-08	ACGTABREMOTIFA2OSEM, ABRELATERD1, ACGTATERD1, GADOWNAT	ABA, GA, and light responses
221	UD	ATGTCGG	2.88E-07	8.44E-07	LTRECOREATCOR15	Cold, dehydration responses
213	UD	CCGACA	3.45E-07	1.73E-08	LTRECOREATCOR15	Cold, dehydration responses
223	UD	GCCGACA	2.08E-06	1.13E-06	LTRECOREATCOR15, DRECRTCOREAT, CBFHV	Cold, dehydration responses
219	UD	GTCGGCA	2.20E-06	5.39E-07	LTRECOREATCOR15, DRECRTCOREAT, CBFHV	Cold, dehydration responses
41	DS	GAAAAATA	2.26E-06	1.91E-06	GT1CONSENSUS, GT1GMSCAM4	Stress and light responses
212	UD	CCGAC	2.52E-06	1.62E-08	LTRECOREATCOR15	Cold, dehydration responses
147	DU	GTTTTTTA	3.61E-06	2.20E-06		
61	DS	TATAATAA	4.18E-06	1.51E-05		
229	UD	ATGTCGGC	5.88E-06	8.31E-06	LTRECOREATCOR15, DRECRTCOREAT, CBFHV	Cold, dehydration responses
42	DS	TATAATA	6.52E-06	1.95E-06		
224	UD	AGCCGCC	6.66E-06	2.77E-06	AGCBOXNPGLB, GCCCORE	Ethylene and other responses
311	US	GGTGAAC	1.05E-05	1.58E-06	GTGANTG10	Pollen expression
55	DS	AATTATTA	1.69E-05	9.17E-06	POLASIG3	Polyadenylation-like motif
210	UD	AATATCT	2.13E-05	1.12E-09	GATABOX, ROOTMOTIFTAPOX1, EVENINGELEMENTLIKE^∗^	Cold, light, and circadian responses
264	UD	GCCGCC	2.24E-05	1.03E-04	GCCCORE	Ethylene and other responses
297	UD	CCGTC	2.45E-05	1.12E-03		
142	DU	AAAATAAC	2.52E-05	8.32E-08	TATABOX5	TATA-box motif
69	DS	AAGTTTAT	2.74E-05	2.56E-05		
44	DS	AATTATAT	3.01E-05	3.37E-06		
161	DU	AGTAAAAA	3.13E-05	1.97E-05	CACTFTPPCA1	Widely distributed *cis*-acting element
245	UD	CATGTCGG	3.70E-05	5.14E-05	LTRECOREATCOR15	Cold, dehydration responses
118	DS	GGTAAAA	3.96E-05	3.21E-04	GT1CONSENSUS	Stress and light responses
53	DS	GTATTTTA	4.51E-05	7.81E-06		
231	UD	GTCGGCAA	4.55E-05	1.84E-05	LTRECOREATCOR15, DRECRTCOREAT, CBFHV	Cold, dehydration responses
230	UD	AAGCCGCC	4.56E-05	1.26E-05	AGCBOXNPGLB, GCCCORE	Ethylene and other responses
244	UD	CCGACAC	5.00E-05	5.06E-05	LTRECOREATCOR15	Cold, dehydration responses
153	DU	AATCATGG	5.62E-05	7.96E-06	ARR1AT	Cytokinin response
261	UD	CGAGGATA	5.69E-05	9.10E-05	GATABOX, MYBST1	Light and MYB responses
63	DS	CTAGTCGC	6.79E-05	1.95E-05		
265	UD	ACCGT	7.03E-05	1.13E-04		
258	UD	CACGCCA	7.81E-05	7.29E-05		
21	DS	ATATAAT	8.23E-05	9.13E-10		
207	SU	CGTAC	8.24E-05	8.29E-04	CURECORECR	SBP response
144	DU	AAATATTT	8.25E-05	1.61E-07	ROOTMOTIFTAPOX1	Starch degradation gene expression
64	DS	AAATAATA	8.63E-05	1.98E-05	POLASIG3, TATABOX5	Polyadenylation-like and TATA motifs
12	DD	AACGAC	8.76E-05	1.96E-04		Auxin response
105	DS	GTTAAAAA	9.57E-05	1.58E-04		
263	UD	GCCGCCC	9.64E-05	9.74E-05	GCCCORE	Ethylene and other responses
96	DS	ACCGCACG	1.03E-04	1.33E-04		
108	DS	AAACTTTA	1.16E-04	1.68E-04	DOFCOREZM, NTBBF1ARROLB, TAAAGSTKST1	Auxin and Dof responses
243	UD	AGGACACG	1.18E-04	5.01E-05		
155	DU	TAATAAAA	1.20E-04	1.01E-05	POLASIG1	Polyadenylation-like motif
250	UD	ACGTGTC	1.23E-04	6.25E-05	ACGTABREMOTIFA2OSEM, ABRELATERD1, ACGTATERD1, GADOWNAT	ABA, GA, and light responses
59	DS	ATAAAAAT	1.29E-04	1.12E-05	SEF4MOTIFGM7S	Widely distributed *cis*-acting element
114	DS	CCGCACG	1.30E-04	2.33E-04		
181	DU	ACGTGAT	1.34E-04	1.79E-04	GTGANTG10, ABRELATERD1, ACGTATERD1, RHERPATEXPA7	ABA, cytokinin, and light responses

*The motif number can be used to find additional motif information in Supplementary Data File 1. ‘Pattern’ is the gene expression pattern, where the first letter indicates the direction of change from paradormancy to endodormancy (U = up-regulated, D = down-regulated, S = same or no change), and the second letter indicates the direction of change from endodormancy to ecodormancy. For example, a pattern of ‘DS’ indicates that the gene was down-regulated from paradormancy to endodormancy, and then did not change from endodormancy to ecodormancy. ‘Seq p-value’ is the random probability of finding the observed number of genes with at least one sequence motif. ‘Motif p-value’ is the random probability of finding the observed mean number of motifs per sequence. PLACE sites are the names of related motifs based on a search of the Database of Plant Cis-acting Regulatory DNA Elements (http://www.dna.affrc.go.jp/PLACE/index.html). Non-PLACE site names are designated with an asterisk.*

## Discussion

### Rationale for the Classification of Dormancy Treatments

We classified the monthly time points into three dormancy treatments based on previous research on *P. trichocarpa* and other cottonwoods, and by grouping the monthly samples based on patterns of gene expression. The first sample was classified as ‘paradormant’ because black cottonwood shoots were still elongating on August 1. Furthermore, in eastern cottonwood, endodormancy is not evident until 2–3 weeks after SD-induced bud set, and does not peak until about 7 weeks after bud set (Howe et al., 1999). The induction of endodormancy seems to progress in a similar fashion in black cottonwood (Frewen et al., 2000; Chen et al., 2002). The November and December samples were classified as ‘endodormant.’ As noted above, SD-induced endodormancy peaked about 7 weeks after SD-induced bud set in eastern cottonwood, and was readily measurable for the next 4 weeks (Howe et al., 1999). This would place peak endodormancy somewhere between our November 1 and December 1 collection dates. Information on the release of endodormancy is also available from experiments on an *F*_2_ population of hybrids between black cottonwood and eastern cottonwood (Chen et al., 2002). Based on these data and other research on balsam poplar (Farmer and Reinholt, 1986), we classified the February and March samples as ‘ecodormant.’

### Differential Expression among Dormancy States

During the transition from paradormancy to endodormancy, most of the differentially expressed genes (*n* = 913; 67%) were down-regulated. In contrast, during the transition from endodormancy to ecodormancy, the two largest groups of genes (*n* = 513 and 519; 38% each) were those that were either up-regulated or did not change. These patterns are consistent with the lower cell division and metabolic activity that occurs during endodormancy. Below, we focus on genes that had opposite changes in expression between the two dormancy transitions (*n* = 604; 44%). That is, genes that were clearly expressed at either higher or lower levels during endodormancy compared to the other two dormancy states. Finally, the smaller number of genes whose expression was atypically higher during endodormancy were of particular interest (*n* = 225; 17%).

### Differential Expression of Chromatin-associated Genes

#### Overview

Large-scale changes in chromatin are associated with plant developmental changes and responses to the environment. These include (1) covalent modifications to histones or DNA and (2) non-covalent remodeling of chromatin, including changes in nucleosome position or stability, and substitution of one histone type for another (Gentry and Hennig, 2014). Several transcriptomic, physiological, and genetic studies have implicated chromatin modifications and remodeling in the regulation of endodormancy (Ruttink et al., 2007; Horvath et al., 2008, 2010; Karlberg et al., 2010). We found additional support for this link, identifying differentially expressed gene sets associated with ‘histone,’ ‘histone methylation,’ ‘chromatin remodeling,’ ‘DNA methylation,’ ‘cytosine methylation,’ and ‘maintenance of DNA methylation.’ The expression of other gene sets and individual genes are discussed in more detail below.

#### RNA-directed DNA Methylation (RdDM)

We observed many changes in genes and gene sets associated with transcriptional gene silencing (TGS) via RNA-directed DNA methylation (RdDM). RdDM is a gene silencing process that is regulated by the methylation and demethylation of DNA at target loci. In general, RNA-DEPENDENT RNA POLYMERASE (RDR) copies single-stranded transcripts into double-stranded RNAs (dsRNAs) that are then processed by DICER-like (DCL) proteins into short interfering RNAs (siRNAs). These siRNAs subsequently associate with ARGONAUT (AGO) proteins to form RNA-induced silencing complexes (RISCs) that mediate DNA methylation and TGS (Matzke and Mosher, 2014). In *Arabidopsis*, the ‘canonical’ RdDM pathway involves RDR2, DCL3, and AGO4 (Matzke and Mosher, 2014). Gene silencing is complicated because post-transcriptional gene silencing (PTGS) involves similar machinery, and the two processes (TGS and PTGS) interact (Matzke and Mosher, 2014). The genes typically associated with PTGS are RDR6, DCL2, DCL4, and AGO1 (Matzke and Mosher, 2014). After TGS is established, DNA methylation and gene silencing can be maintained by CMT3 (Matzke and Mosher, 2014). Differentially expressed genes and gene sets included genes that seem to encode RDR (RDR1, RDR6), DCL (DCL1, DCL2), ARGONAUT (AGO4, AGO7), and CHROMOMETHYLASE 3 (CMT3). Because of the complexity and similarities of the TGS and PTGS pathways, we did not attempt to link particular *Populus* genes and gene sets to each of these two gene silencing pathways (i.e., TGS versus PTGS). Endodormancy related changes in RdDM pathways have been observed in other perennial species. For example, a gene similar to *Arabidopsis AGO4* and a gene that may be functionally similar to *DCL4* were also down-regulated during endodormancy in leafy spurge (Horvath et al., 2008).

#### Histone Modifications

An assortment of gene sets involved in histone modifications were differentially expressed, including neighbors of ‘histone’ and ‘polycomb complex.’ In *Arabidopsis*, the Polycomb Repressive Complex 2 (PRC2) participates in stable gene silencing. PRC2 methylates histone H3, resulting in the repression of gene expression (Kim et al., 2012). For example, PRC2 controls flowering via histone methylation of *FT* chromatin (Jiang et al., 2008) and represses *FLOWERING LOCUS C (FLC)* during vernalization (De Lucia et al., 2008). Other differentially expressed gene sets included neighbors of ‘SWN,’ ‘FIE,’ and ‘PKL.’ In *Arabidopsis*, *SWN* and *FIE* encode core components of PRC2 (Deng et al., 2013), whereas *PKL* encodes a DNA-binding helicase which seems to associate with PRC2 target loci to enhance histone modification (Zhang et al., 2012). Interestingly, aspen genes similar to *FIE* and *PKL* were up-regulated during SD-induced bud set (Ruttink et al., 2007). Although genes that presumably encode ‘neighbors’ of FIE and PKL were differentially expressed in our study, the genes themselves were not. That is, we did not see differential expression of two *PKL*-like genes (Potri.006G262200 and Potri.018G021100) and one *FIE*-like gene (Potri.001G417300) in our study.

‘Binding partners of DDB1A’ was another differentially expressed gene set that seems to be associated with gene silencing via PRC2. In *Arabidopsis*, DDB1A is a component of the CULLIN 4 (CUL4)/DDB1 ubiquitin ligase complex that functions in a wide array of plant processes, including flowering, photomorphogenesis, and parental imprinting (Hou et al., 2014). The CUL4/DDB1 complex seems to interact with histone tails to repress the transcription of genes involved in photomorphogenesis (Benvenuto et al., 2002), and an association between CUL4/DDB1A and PRC2 seems to regulate flowering time in *Arabidopsis* (Dumbliauskas et al., 2011; Pazhouhandeh et al., 2011). The differential expression of a ‘DET1’ gene set provides a specific link to endodormancy. The DET1 protein interacts with CONSTITUTIVE PHOTOMORPHOGENIC 10 (COP10) and the CUL4/DDB1 complex to regulate responses to light and temperature (Delker et al., 2014).

Gene activation and silencing also involve histone acetylation and deacetylation. In general, histone acetylation is associated with gene activation, whereas deacetylation is associated with gene silencing. Two differentially expressed gene sets were associated with histone deacetylases—HDA6 and HDA19 (also known as HD1). HDA6 is a histone deacetylase that has been identified as a component of the *Arabidopsis* RdDM machinery (To et al., 2011a). In particular, deacetylation of histone H3 seems to be important for the subsequent methylation of histone H3 described above (To et al., 2011a). In *Arabidopsis*, HDA6 is involved in the regulation of flowering, senescence, leaf development, the circadian clock, and responses to salt stress, ABA, and JA (Wu et al., 2008b; Chen et al., 2010; To et al., 2011b; Liu et al., 2014), whereas HDA19 regulates seed maturation and flower development (Liu et al., 2014). Other differentially expressed genes and gene sets are connected to these histone deacetylases. For example, JAZ proteins recruit HDA6 to inhibit JA signaling (Zhu et al., 2011).

One of the strongly differentially expressed genes (Potri.014G189400; DNG5) is a putative homolog of the vertebrate gene *MBD4 (METHYL-CPG-BINDING DOMAIN 4;* Ramiro-Merina et al., 2013). MBD proteins may recruit histone deacetylases such as HDA6—thereby acting as the ‘bridges’ between DNA methylation and histone deacetylation (Liu et al., 2012).

Finally, two *SPT*-like genes had atypical patterns of expression—being strongly up-regulated from paradormancy to endodormancy, and then down-regulated from endodormancy to ecodormancy. *Arabidopsis* SPT5-2 is part of the SPT4/SPT5 transcript elongation factor that seems to link transcription elongation, histone modification, and chromatin remodeling in yeast and *Arabidopsis* (Hartzog and Fu, 2013; Durr et al., 2014). Furthermore, an *Arabidopsis SPT5* homolog (*KTF1/RDM3/SPT5*-like) has been linked to AGO4-mediated gene silencing (Karlowski et al., 2010; Hartzog and Fu, 2013). A *Populus* gene similar to *Arabidopsis SPT6L* was also atypically expressed at higher levels during endodormancy—and *Arabidopsis SPT6L* seems to interact with AGO proteins to regulate embryo development (Gu et al., 2012). Thus, it is curious that a gene similar to *AGO4* (Potri.001G219700) was down-regulated during endodormancy in our study and in other plants (Horvath et al., 2008).

#### Other Chromatin-associated Genes

Three other genes were clearly expressed at lower levels during endodormancy. The first gene, Potri.004G087500, is similar to *Arabidopsis HMGA*. HMGA proteins interact with A/T-rich DNA, altering the chromatin structure and transcription of their target genes (Reeves, 2010). The second gene, Potri.008G155400, is similar to *Arabidopsis SILENCING DEFECTIVE 3* (*SDE3*), which is more clearly associated with PTGS. The third gene, Potri.T029800, is similar to an *Arabidopsis* gene *(ALPHA/BETA HYDROLASE F4*; *ABHF4*) that is associated with the alpha/beta-hydrolase superfamily of proteins with unknown function.

#### Roles of Chromatin-associated Genes in Endodormancy Induction and Release

Dormancy transitions were accompanied by changes in multiple genes associated with DNA methylation (e.g., via RdDM) and histone modifications (e.g., via PRC2). However, although we expected to see increased expression of TGS components during endodormancy—the opposite was true—most chromatin-associated genes and gene sets were expressed at lower levels during endodormancy. One explanation is that reduced gene silencing may activate genes that positively induce and maintain endodormancy. *DAM* genes, for example, which seem to participate in the induction and maintenance of endodormancy, show reduced histone methylation (Horvath et al., 2008; Leida et al., 2012). Second, reduced expression of chromatin-associated genes may simply reflect the lower cell division and metabolic activity that occurs during endodormancy. Most of the other non-chromatin-associated genes were also expressed at lower levels during endodormancy. Third, subtle changes in dormancy-specific TGS may have been swamped by other processes. We compared paradormant buds (not actively growing meristems) to endodormant buds, and only subtle differences in the complement of active and silenced genes may exist between these two dormant states. Finally, the transition from paradormancy to endodormancy may involve a transient increase in gene silencing activity—an increase that we missed with our 1–2 month sampling interval.

### Differential Expression of Transcription Factor Genes

#### Ethylene-associated Transcription Factors

We found substantial evidence for the differential expression of genes associated with ethylene responses, including transcription factors. Gene sets associated with EIN3 and EIL1 (EIN3-LIKE 1) were expressed at higher levels during endodormancy. The EIN3/EIL1 transcription factors act downstream of the signaling protein EIN2 to positively regulate the ethylene response pathway, including leaf senescence (Kim et al., 2014). The induction of ethylene responses during endodormancy is also supported by the differential expression of gene sets associated with the ETHYLENE-RESPONSIVE ELEMENT BINDING PROTEIN (EREBP), EIN2 membrane protein, EIN4 ethylene receptor—as well as individual genes that encode ETHYLENE RESPONSE FACTOR (ERF) proteins, which belong to the APETALA 2 (AP2)/EREBP family of transcription factors. Five of the six ERFs described in **Figure 4** seemed to be expressed at higher levels during endodormancy. Although the details differ, ERF genes have also been implicated in bud dormancy in hybrid aspen and Japanese apricot (Rohde et al., 2007; Zhong et al., 2013). Kim et al. (2014) proposed that AtNAP and other ‘senescence-associated’ NAC transcription factors act downstream of EIN2, and genes that seem to encode AtNAP and other NAC proteins were differentially expressed (**Figure 4**). Gene sets associated with EIN2, an ethylene signaling component, and EIN4, an ethylene receptor, were also expressed at higher levels during endodormancy. Changes in ethylene-associated transcription factors and other genes support broader physiological evidence that ethylene has an important functional role in bud dormancy (Ruonala et al., 2006; Rohde et al., 2007). Finally, our results suggest that JA interacts with ethylene to regulate bud dormancy. This is in agreement with indications of JA signaling during dormancy transitions observed in leafy spurge and Japanese apricot (Horvath et al., 2008; Zhong et al., 2013). In *Arabidopsis*, EIN3/EIL is a ‘key integration node’ that integrates signaling by ET and JA (Zhu et al., 2011), and gene sets associated with CORONATINE INSENSITIVE 1 (COI1) and JASMONATE ZIM-DOMAIN 10 (JAZ10) were differentially expressed. COI1 is a JA receptor and JAZ10 is a transcriptional repressor (Zhu et al., 2011). Furthermore, HDA6, a histone deacetylase involved in gene silencing (discussed above), interacts with JAZ proteins and COI1 to repress EIN3/EIL1-mediated transcription and JA signaling (Zhu et al., 2011).

#### WRKY DNA-binding Domain Transcription Factors

WRKY transcription factors, which contain the WRKY DNA-binding domain, have been described as ‘major hubs’ in abiotic stress signaling (Tripathi et al., 2014). Several genes associated with WRKY transcription factors were differentially expressed. These include gene sets associated with *Arabidopsis* WRKY, WRKY33, and WRKY70 (Supplementary Tables S5 and S6), and individual genes similar to *Arabidopsis WRKY5*, *WRKY6*, *WRKY27*, *WRKY40*, and *WRKY33* (**Figure 4**; Supplementary Data File 1). There seem to be about 100 WRKY genes in *P. trichocarpa*, many of which are induced by SA, JA, cold, drought, salinity, or wounding (He et al., 2012; Jiang et al., 2014). Thus, the WRKY transcription factors provide one potential link between dormancy-associated gene expression and the phytohormones JA and SA (discussed below).

#### Cold-responsive Transcription Factors

Another gene set that was strongly associated with endodormancy was ‘Neighbors of RHL41’ (ZINC FINGER PROTEIN 12, ZAT12). ZAT12 is one of the transcription factors induced very quickly after exposure to cold temperatures (Vogel et al., 2005; Park et al., 2015). Other genes encoding ‘first wave’ transcription factors were differentially expressed in our study as well (e.g., *WRKY33* and *ZAT10/STZ*; **Figure 4**), but others were not, including genes encoding the C-REPEAT BINDING FACTORS (CBFs). Induction of *ZAT12* leads to the induction of some cold-responsive (COR) genes, and the repression of others; and overexpression of *ZAT12* leads to enhanced freezing tolerance (Vogel et al., 2005). A gene that seems to encode a ZAT10/STZ transcription factor was expressed at higher levels during endodormancy (**Figure 4**). In *Arabidopsis*, the expression of *ZAT10/STZ* is regulated by cold, drought, and salt; and overexpression of *ZAT10/STZ* enhances drought tolerance (Sakamoto et al., 2004). Although *CBF* genes are clearly involved in acclimation to cold and drought (Thomashow, 2010), *CBF*-like genes were not differentially expressed in our study. Nonetheless, CBF binding motifs were significantly overrepresented in the promoters of genes that were up-regulated during endodormancy (discussed below). In a similar study of aspen, none of the four *CBF*-like genes was up-regulated during 5 weeks of SDs (Karlberg et al., 2010). Although another *CBF*-like gene was up-regulated for a short time, it was then down-regulated to almost the original level during the endodormant period. In our study, we may have missed transient increases in *CBF* gene expression because of the 1–2-month sampling interval we used.

#### Dormancy-associated Transcription Factors

Genes encoding other transcription factors were expected to be differentially expressed. For example, *DORMANCY ASSOCIATED MADS-BOX (DAM)* genes are putative transcription factors found in perennial plants that have been directly linked to vegetative endodormancy. They are similar to two genes, *SHORT VEGETATIVE PHASE (SVP)* and *AGAMOUS-LIKE 24 (AGL24)*, that encode transcription factors regulating flowering time in *Arabidopsis*. In peach (*Prunus persica*), a deletion of *DAM* genes resulted in trees that were unable to become endodormant, and *DAM* expression is enhanced during endodormancy in several perennial species (Horvath et al., 2010; Jiménez et al., 2010). In our study, several *DAM*-like (*SVP*-like) genes were differentially expressed (Potri.005G155700, Potri.017G044500, and Potri.002G105600), but they were down-regulated during endodormancy, unlike the *DAM* genes in leafy spurge and peach (Horvath et al., 2010; Jiménez et al., 2010). A different *DAM*-like gene (Potri.007G010800) was up-regulated during the induction of endodormancy in hybrid aspen (Ruttink et al., 2007), and strongly down-regulated in early flushing trees that were overexpressing *EARLY BUD-BREAK 1* (*EBB1*; Yordanov et al., 2014). However, this gene was not differentially expressed in our study. Finally, one gene (Potri.001G328400) was highly up-regulated in our December and February samples—but these differences were not significant among months or dormancy states (FDR *p*-value = 0.07 to 0.11). This gene encodes an unusual truncated transcript, which is reminiscent of the truncated splice variant of the endodormancy-induced *DAM* transcript in leafy spurge (Horvath et al., 2013). Overall, these disparate results provide limited insight into the connections between endodormancy and *DAM*-like genes in *Populus*.

#### Other Transcription Factors

Other gene sets associated with transcription factors were strongly down-regulated during endodormancy. JLO, SEU, RPL, and ARF2 seem to have various roles in auxin signaling, including organization of the shoot apical meristem and organ development (Franks et al., 2006; Sluis and Hake, 2015). This suggests they could be involved in the formation or development of new leaf primordia. If so, their patterns of expression (i.e., lower expression during endodormancy) are consistent with the cessation of primordia initiation and development that occurs during dormancy induction.

Genes that seem to encode MYB transcription factors were also common among the genes that were down-regulated from paradormancy to endodormancy (**Figure 4**). Given that the *MYB* family is very large, and endodormancy is associated with a general reduction in metabolic activity, the significance of these changes is uncertain. Nonetheless, MYB14 represses the CBF regulon in *Arabidopsis*, (Chen et al., 2013), but we did not see differential expression of the *Populus CBF/DREB* genes in our study (discussed above).

Finally, genes encoding other flowering-associated transcription factors were also differentially expressed, including *SQUAMOSA PROMOTER BINDING PROTEIN-LIKE (SPL)* and *SUPPRESSOR OF OVEREXPRESSION OF CONSTANS 1 (SOC1)*. These and other flowering-associated genes are discussed in more detail below.

### Differential Expression of Phytohormone-associated Genes

#### Auxin-associated Gene Expression

Auxin-associated genes were generally down-regulated during endodormancy, with most changes expected to lead to a reduction in auxin signaling. This, and other more specific changes in gene expression, suggests that auxin signaling undergoes important changes during dormancy transitions. The first step in auxin signaling involves an interaction between auxin, an auxin receptor such as TIR1, and AUXIN/INDOLE 3-ACETIC ACID (Aux/IAA) proteins. This interaction ultimately leads to the degradation of the Aux/IAA proteins, which normally repress ARF transcription factors. The reduction in Aux/IAA leads to enhanced transcription of auxin-inducible genes by ARF and downstream auxin responses (Korasick et al., 2014). In our study, genes that encode binding partners of TIR1 were down-regulated from paradormancy to endodormancy, and a gene that was strongly up-regulated (Potri.010G078400) is similar to the *Arabidopsis* gene that encodes IAA4. This latter change, in particular, is consistent with a reduction in auxin signaling and auxin responses during endodormancy. Furthermore, gene sets associated with three ARF transcription factors were differentially expressed between dormancy states. In *Arabidopsis*, ARFs are also negatively regulated by miRNAs, including *miR160* (Rhoades et al., 2002; Paponov et al., 2009). In our study, the gene set associated with *miR160A* was significantly down-regulated from paradormancy to endodormancy (Supplementary Table S5). Likewise, genes that encode neighbors and targets of *miR393A* and *miR393B* were differentially expressed between paradormancy to endodormancy. This miRNA seems to negatively regulate the gene encoding TIR1 (Liu et al., 2009). Corresponding changes in auxin and auxin-associated gene expression have been found in other species. In silver birch, auxin declined during SD-induced endodormancy (Li et al., 2003), and auxin-associated genes were down-regulated during endodormancy in the cambial meristem of *Populus* (Baba et al., 2011) and the buds of leafy spurge (Horvath et al., 2008). Our results concur, and because of its atypical pattern of expression, point to a particularly important role for the *IAA4*-like gene (Potri.010G078400).

Down-regulation of auxin transport also seems to occur during endodormancy. For example, genes similar to two *Arabidopsis* genes involved in auxin transport were down-regulated from paradormancy to endodormancy. The first gene (Potri.002G087000) is similar to a gene that encodes the auxin influx carrier, LAX3 (LIKE AUX3). The second (Potri.006G123900) is similar to a gene that encodes an ATP-BINDING CASSETTE (ABC) transporter that regulates basipetal auxin transport. Consistent with these changes, two other genes (Potri.012G047200 and Potri.016G035300) were up-regulated from endodormancy to ecodormancy, both of which are similar to the *Arabidopsis PIN1* (*PIN-FORMED 1*), which encodes a putative auxin eﬄux transporter (Sluis and Hake, 2015).

Finally, changes in the expression of genes associated with the synthesis of phenylpropanoids and flavonoids may affect auxin responses. As discussed below, these genes were mostly down-regulated during endodormancy, which could *enhance* auxin transport, but also destabilize auxin levels by increasing auxin oxidation (Brown et al., 2001; Buer and Muday, 2004; Peer et al., 2013).

#### Ethylene-associated Gene Expression

The ethylene-associated gene set was up-regulated during endodormancy, which was opposite from what we observed for auxin and BR. However, this gene set included a mix of genes with positive and negative effects on ethylene signaling. For example, genes that seem to encode negative (CTR1) and positive (ERF5) regulators of ethylene signaling were both up-regulated during endodormancy. Although this seems counterintuitive, genes similar to *CRT1* and *ERF* are induced by exogenous ethylene, demonstrating some degree of coordinate regulation (Vahala et al., 2013; Zou et al., 2014). Other related gene sets were up-regulated during endodormancy, including those associated with the ethylene receptor EIN4 and the transcription factors EIN2 and EIN3. Up-regulation was also observed for multiple genes that seem to encode ERF transcription factors, at least some of which have been specifically associated with responses to ethylene. Ethylene has been implicated in bud endodormancy, perhaps in concert with ABA. Ruttink et al. (2007), for example, concluded that ethylene and ABA act sequentially during SD-induced bud dormancy in *Populus*. Although they emphasized the transient nature of increases in ethylene-associated gene expression, we observed up-regulation of multiple ethylene-associated genes over a two-month period under natural conditions. Interacting roles for ethylene and ABA during endodormancy induction and release have also been reported in birch and grape, which is consistent with our results (Ruonala et al., 2006; Ophir et al., 2009; Zheng et al., 2015).

#### GA- and ABA-associated Gene Expression

Perhaps the most consistent associations between bud endodormancy and phytohormones are the opposing changes in GA and ABA—GA levels tend to decline and ABA levels increase early in the induction of endodormancy (reviewed in Olsen, 2010). For example, SD-induced down-regulation of GA-20-oxidase reduces the levels of active GAs in *Salix* and *Populus* (reviewed in Olsen, 2010), and up-regulation of GA-2-oxidases may lead to GA inactivation (Zawaski and Busov, 2014). However, we did not observe differential expression of any genes that seem to encode GA-20-oxidase or GA-2-oxidase, and the changes in other genes associated with GA and ABA were contrary to expectations. It is possible that transient changes in GA- and ABA-related gene expression were missed due to our monthly sampling scheme. Most previous analyses focused on the early stages of dormancy induction, often focusing on changes in response to SDs. Our results suggest that GA and ABA have (at most) modest roles in the maintenance of endodormancy *per se*. For example, *Populus* trees genetically engineered to underexpress or overexpress the *ABA INSENSITIVE 3* (*ABI3*) transcription factor still became endodormant under SDs (Ruttink et al., 2007), and other studies suggest that ABA is primarily involved in the formation of the dormant bud, not in the maintenance of endodormancy (reviewed in Olsen, 2010).

#### Brassinosteroid-associated Gene Expression

Brassinosteroid has non-redundant roles as a “potent” growth-promoting phytohormone (Schröder et al., 2014). Although clear involvement in bud endodormancy has not been previously established, changes in the expression BR-related genes point in this direction. First, the BR hormone-associated gene set, ‘Binding partners of BES1,’ was down-regulated during endodormancy. BES1 is a BR-regulated transcription factor, and low BES1 signaling helps ensure stem cell quiescence in plants (Vilarrasa-Blasi et al., 2014). Second, three genes expected to enhance BR signaling were all down-regulated during endodormancy (i.e., compared to paradormancy or ecodormancy). Two of these genes are similar to an *Arabidopsis* gene (*CBB1*) that encodes a sterol reductase involved in the early steps of BR synthesis (Choe et al., 1999). The third gene is similar to *BRASSINOSTEROID SIGNALING KINASE 2 (BSK2)*, which belongs to a family of genes that encode positive regulators of BR signaling (Sreeramulu et al., 2013). Thus, it appears that down-regulation of BR biosynthetic and signaling genes may help maintain the reduced cell division associated with endodormancy. Because of crosstalk between BR and SA signaling (Divi et al., 2010), there may be a link between the changes in BR-associated genes described above, and the changes in SA associated genes described below.

#### Salicylic-acid-associated Gene Expression

During the transition from paradormancy to endodormancy, we found clear evidence for changes in the expression SA-associated genes. Genes with the strongest patterns of differential expression include three genes similar to *Arabidopsis PAL1*, which encodes a key enzyme in SA biosynthesis. All three genes were down-regulated from paradormancy to endodormancy. Although connections between SA and bud endodormancy have not been widely reported, SA promoted endodormancy release in grape (i.e., as measured by H_2_O_2_ production; Or et al., 2000). Nonetheless, changes in SA-associated genes may be more closely related to general changes in the phenylpropanoid pathway, rather than endodormancy *per se*—such as changes in gene sets associated with anthocyanin and flavonoid biosynthesis (Nugroho et al., 2002). Although genes associated with proanthocyanin production have been associated with seed dormancy (Debeaujon et al., 2000), corresponding gene sets were mostly down-regulated in our study, including gene sets associated with GLABRA 3, ENHANCER OF GLABRA 3, TRANSPARENT TESTA 2 (TT2), TT8, TRANSPARENT TESTA GLABRA 1 (TTG1), and CAPRICE (Supplementary Tables S5 and S6). Although SA may contribute to endodormancy, the changes in phenylpropanoid and SA gene expression may have no direct role in endodormancy.

#### Jasmonic-acid-associated Gene Expression

We found consistent evidence for down-regulation of many JA-associated genes and gene sets from paradormancy to endodormancy, including genes involved in JA synthesis and signaling. Because enhanced JA signaling is associated with responses to stress and decreased growth, many of these changes contradict the expected roles that JA might have during endodormancy. Given that these changes coincide with broader reductions in gene expression during endodormancy, their relevance is uncertain. Nonetheless, because JAZ proteins repress JA signaling, the down-regulation of a gene similar to *JAZ12* (Potri.018G047100) could lead to increased JA-repressed growth during endodormancy. Finally, because JA regulates anthocyanin accumulation through COI, down-regulation of JA signaling could contribute to the reduction in in phenylpropanoid and SA-associated gene expression described above. Additional efforts are needed to identify the mechanisms underlying the seemingly contradictory responses in JA signaling and synthesis.

#### Cytokinin-associated Gene Expression

We found limited evidence to suggest that changes in cytokinin (CK) gene expression are important during endodormancy induction and release. Although one gene set, ‘Neighbors of cytokinin,’ and three individual genes were all down-regulated from paradormancy to endodormancy, these changes mirror the broader reductions in gene expression that occurred during endodormancy. Therefore, their relevance is uncertain. On balance, however, these changes are consistent with the hypothesis that cytokinin acts as an antagonist of auxin-mediated apical dominance by promoting the outgrowth of paradormant buds (Mueller and Leyser, 2011).

### Flowering Genes and Processes are Associated with Vegetative Bud Dormancy

We previously presented a general model for endodormancy involving *FT*-based regulatory networks analogous to the networks that regulate flowering (reviewed by Horvath, 2009). Consistent with this model, our current results suggest an important role for genes similar to *SPL* in *Populus*. In *Populus*, *FT2* inhibits growth cessation and bud set. Thus, down-regulation of *FT2* seems to be an important early step in the induction of endodormancy (Bohlenius et al., 2006; Ruonala et al., 2008; Hsu et al., 2011). In *Arabidopsis*, photoperiodic regulation of *FT* involves a network of *miR156*, SPL proteins, *miR172*, and a set of AP2-like transcription factors, including AP2, TOE1-3 (TARGET OF EARLY ACTIVATION TAGGED 1-3), SCHLAFMÜTZE (SMZ), and SCHNARCHZAPFEN (SNZ; Aukerman and Sakai, 2003; Schmid et al., 2003; Jung et al., 2007). A model has emerged in which SPLs positively regulate *miR172*, which normally represses AP2, TOEs, SMZ, and SNZ (Wellmer and Riechmann, 2010). Thus, up-regulation of SPLs represses these AP2-like transcription factors, leading to an increase in *FT* expression and the promotion of flowering. In our study, eight *SPL*-like genes were down-regulated from paradormancy to endodormancy. By analogy to the flowering pathway described above, down-regulation of *SPL* genes should lead to down-regulation of *miR172*, up-regulation of genes similar to *AP2, TOE*, *SMZ*, and *SNZ*, repression of *FT2*, and ultimately, growth cessation, bud set, and endodormancy. However, despite the consistent down-regulation of *SPL*-like genes in our study, two other observations contradict this simple model. First, three *DAM*-like (*SVP*-like) genes were unexpectedly down-regulated in our study, which is opposite of what has been seen in other perennial plants (discussed above). Furthermore, because SVP negatively regulates *miR172* in *Arabidopsis* (Cho et al., 2012), down-regulation of *DAM*/*SVP* genes in *Populus* is ultimately expected to increase, rather than decrease, the expression of *FT2*. However, SVP is not uniformly up-regulated during flower development. In *Arabidopsis*, *SVP* is repressed in early flower development to prevent flower reversion, and in late flower development to allow the activation of *SEPALATA3* (Lee and Lee, 2010). Therefore, our results may indicate that the timing of *DAM/SVP* expression is important in *Populus* as well. Second, *FT2* itself was not differentially expressed. However, this was not surprising because *FT2* expression decreases dramatically after only a few SD (Resman et al., 2010). Therefore, the longer-term changes in *SPL* gene expression that we observed may help keep the expression of *FT2* and other flowering-related genes at already low levels, rather than being the direct, early cause of *FT2* down-regulation.

Like *FT*, *SOC1* (also known as *AGL20*) is considered a major integrator of flowering signals in *Arabidopsis*. In our study, two *SOC1*-like genes and the gene set “Binding partners of AGL20” were down-regulated from paradormancy to endodormancy, and similar results were observed in other studies of *Populus* (Ruttink et al., 2007) and leafy spurge (Horvath et al., 2008; data not shown). *SOC1* expression generally promotes flowering in *Arabidopsis*. More specifically, studies in *Arabidopsis* (Tao et al., 2012) show that SOC1 directly down-regulates *AP2*, *TOE1*, and *SMZ*, which are downstream targets of *SPLs* and *miR172* (described above). Finally, allelic variation in a *SOC1* homolog has been linked to dormancy in apricot (Trainin et al., 2013). Given the functional and regulatory similarities between *DAM* and *FLC*, a repressor of *SOC1*, a similar mechanism involving *SOC1*-like genes might regulate vegetative bud endodormancy.

Despite the potential connections between endodormancy and flowering-like genes, the genes described above have been implicated in diverse processes, including endodormancy induction (e.g., photoperiodism), endodormancy release (e.g., chilling), cold acclimation, flowering, and fruit development (Preston and Sandve, 2013). For example, in *Arabidopsis*, there seems to be a feedback loop between cold responses and flowering-time that involves interactions between CBF, SOC1, and FLC (Seo et al., 2009). Therefore, it will be challenging to dissect the functional significance of dormancy-associated changes in expression, particularly for genes that serve as key regulatory hubs.

### Differential Expression of Genes Associated with Bud Set QTL

Rohde et al. (2011b) identified six robust QTL associated with various components of bud set, and then compared the QTL locations to the locations of differentially expressed genes (Ruttink et al., 2007). We conducted a similar analysis using a larger number of genes and updated gene models (*Populus* v3.0). Although hundreds of genes were associated with each QTL, the number of differentially expressed genes ranged from 9 to 24. We then examined relationships between the QTL and the various classes of genes described above (chromatin-associated, transcription factor, and hormone-associated) to identify promising QTL candidates.

We found 14 differentially expressed genes that are reasonable QTL candidates based on their differential expression and putative functions. These include four genes that seem to be involved in chromatin remodeling (e.g., *DCL4*). Other differentially expressed genes seem to be involved in BR synthesis (*CBB1*) or phytohormone signaling via ABA (*PYL*), JA (*JAZ12*), or auxin (*IAA13*). Additional genes are more generally associated with responses to far-red light (*FAR-RED IMPAIRED RESPONSIVE 1*), organization of lateral organ boundaries (*LOB DOMAIN-CONTAINING PROTEIN 21*), and mitotic arrest (*MAD2*). Finally, we also found genes that seem to encode a NAC domain transcription factor associated with leaf senescence, and other PHD and bHLH transcription factors that are not as well characterized. In addition to these candidates, a few other differentially expressed genes with unknown functions should be considered.

Overall, these results suggest that differential expression can be used to reduce a large number of positional candidate genes (>2,000) to a much smaller set of plausible QTL candidate genes. Nonetheless, it would still be challenging to investigate all of these candidates in detail using functional genomics. For example, the genes underlying these QTL may not be differentially expressed, or the changes in expression may be transient. Furthermore, transgenic functional approaches often cause major, poorly timed perturbations that may lead to responses that do not accurately reflect natural gene functions. A combination of approaches including fine-scale mapping, association genetics, analyses of gene expression, and subtle gene perturbations will be needed to understand the roles of the numerous genes that appear to regulate dormancy transitions.

### Upstream Sequence Motifs are Associated with Specific Patterns of Gene Expression

Motifs associated with photoperiodic responses and circadian patterns of gene expression were highly enriched in some gene expression pattern groups. For example, two of the top-ranked motifs match binding sites for two central transcription factors that regulate the circadian clock, LATE ELONGATED HYPOCOTYL (LHY) and CIRCADIAN CLOCK ASSOCIATED 1 (CCA1). The first motif matches the EVENING ELEMENT-LIKE (EEL) motif (AATATCT). The EEL and the EVENING ELEMENT itself (EE, AAAATATCT) are important regulators of circadian clock and cold-responsive genes (Mikkelsen and Thomashow, 2009). The EEL motif was our top-ranked motif, being significantly overrepresented in genes that were up-regulated during endodormancy. As summarized by Hsu and Harmer (2014), most clock components regulate the transcription of genes that contain EE, or are regulated by other clock components through EE in their own promoters. The second motif matches a binding site (AAAAATCA) that is found in the target genes of CCA1 (Maxwell et al., 2003), being enriched in genes that were down-regulated during endodormancy.

Other motifs previously associated with circadian patterns of gene expression were also found, including G-box (CACGTG), I-box core (GATAA), PIF-binding E-box (PBE-box; CACATG), and the CAB2 DET1-ASSOCIATED FACTOR 1 binding site motif (CDA-1; CAAAA). Both the G-box and PBE-box elements are binding sites for PIF3, a transcription factor that interacts with phytochromes A and B (Zhang et al., 2013). Furthermore, the HORMONE UP AT DAWN (HUD) element, which has the same sequence as the PBE-box, is overrepresented in the promoters of phytohormone genes (Michael et al., 2008). We also found many motifs that matched the circadian elements described in Table 1 of Smieszek et al. (2014), only some of which were identified using the PLACE database. Photoperiodic and circadian regulation of endodormancy has been well documented (Howe et al., 1996; Rohde and Bhalerao, 2007; Horvath et al., 2010), and was also highlighted by our GSEA. Gene sets associated with two components of the circadian clock, ZEITLUPE (ZTL) and LIGHT-REGULATED WD 2 (LWD2), were up-regulated during endodormancy. ZTL is a blue light photoreceptor that regulates photoperiodic responses (Kim et al., 2007), and LWD2 encodes a WD (tryptophan and aspartate) protein that contributes to clock function (Wu et al., 2008a).

A second class of overrepresented motifs was broadly associated with responses to cold, dehydration, and ABA. The most prominent of these is the RCCGAC motif, which is the core of the C-repeat (CRT) element, also known as the dehydration responsive element (DRE). The CRT/DRE element is the binding site for CBFs (C-REPEAT BINDING FACTORS), some of the most important transcription factors involved in cold-induced gene expression (Benedict et al., 2006). CBFs regulate gene expression in response to cold and dehydration. Other transcription factors, such as the ABA RESPONSIVE ELEMENT BINDING PROTEIN (AREB), regulate gene expression in response to cold and dehydration in an ABA-dependent manner. These transcription factors bind to ABA responsive elements (ABRE; ACGT core), ABRE-like elements (ABREL; AGCTG), and G-box elements (Mikkelsen and Thomashow, 2009). We found many enriched motifs that contain these core sequences.

We found a number of dehydrin-like genes that were up-regulated from paradormancy to endodormancy, and these genes provide good models for how different combinations of *cis*-regulatory elements can lead to different patterns of gene expression. For example, Zolotarov and Stromvik (2015) identified 14 conserved motifs in 350 dehydrin promoters from 51 plant genomes, many of which were similar to motifs that were overrepresented in our differentially expressed genes, as well as in genes that have been associated with responses to cold, dehydration, ABA, and light.

We also found motifs associated with other phytohormones, including ethylene, auxin, SA, and JA. Among the top-ranked motifs, we found four with the GCC-box core motif (GCCGCC), which serves as a binding site for ethylene-responsive genes (Ohme-Takagi and Shinshi, 1995). These motifs were highly enriched in genes that were up-regulated from paradormancy to endodormancy. This coincides with our GSEA results indicating that ethylene is an important regulator of endodormancy. We also found five motifs that matched two auxin responsive elements (NTBBF1ARROLB and TGA-element; **Table 1**), and all five motifs were enriched in genes that were down-regulated from paradormancy to endodormancy. Again, this supports our GSEA results indicating that auxin related genes were mostly down-regulated from paradormancy to endodormancy. We found a large number of motifs that are associated with responses to cytokinin, but their significance is unclear; we saw no strong trends in cytokinin-related gene expression. The longest matching PLACE motif was CPBCSPOR (TATTAG), which exhibits cytokinin-enhanced protein binding, but the other two PLACE motifs, ARR1AT (NGATT) and RHERPATEXPA7 (KCACGW), are much less specific. We also found a small number of motifs that have been non-specifically associated with JA and SA.

In sum, our analyses of promoter motifs showed clear associations between patterns of endodormancy-related gene expression and two broad classes of genes—those associated the circadian clock and photoperiodic responses, and those associated with phytohormone-mediated responses to cold and dehydration. An understanding of the finer details of gene regulation are complicated by the fact that many of the consensus motifs are short and widely distributed among plant promoters involved in responses to light, biotic and abiotic stresses, and phytohormones. Furthermore, about 45% of the enriched motifs had no assigned functions, suggesting that more work is needed to understand the functions of these motifs and their potential roles in the regulation endodormancy-associated processes. Further insights could be gained by analyses that focus on understanding how the numbers, distributions, and combinations of motifs are associated with genes known to have specific patterns of gene expression across *Populus* species.

## Conclusion

Our work highlights both the conserved nature and the extraordinary complexity of transcriptome changes associated with vegetative dormancy. For example, we confirmed and elaborated upon earlier evidence from studies of chromatin remodeling. We found multiple genes associated with DNA methylation (e.g., via RdDM) and histone modifications (e.g., via PRC2) that were differentially expressed during the induction and release of endodormancy. We identified 19 chromatin-associated genes that were down-regulated during endodormancy, and two genes that were strongly and atypically up-regulated. These latter two genes are similar to *Arabidopsis SPT5-2* and *SPT6L*, which encode proteins described as ‘global’ transcription factors. We also identified links to genes that regulate the onset of flowering, pointing to potentially important roles for genes similar to *SPL*, *DAM/SVP*, and *SOC1*. Differential expression of *SPL* genes corroborates earlier observations and implicates miRNA-associated regulatory pathways in the repression of *FT2* during endodormancy.

A number of surprises emerged from our analyses of phytohormone-related genes. Changes in genes encoding GA-20-oxidase and GA-2-oxidase were not observed, and changes in genes associated with ABA were contrary to expectations. Although we may have missed transient changes associated with short-day-induced bud set, these results suggest that these phytohormones have relatively narrow windows of action. In contrast, we saw clearer evidence for changes in the expression of genes associated with ethylene, auxin, BR, SA, and JA. For example, genes and gene sets that were atypically up-regulated during endodormancy included those associated with responses to ethylene (*EIN3, EBP, ERFs*), and a gene similar to *Arabidopsis IAA4*. However, genes associated with auxin, BR, SA, and JA were mostly down-regulated during endodormancy. Because of the general down-regulation of metabolic activity and gene expression during endodormancy, the biological significance of these changes warrants further study.

Other genes that were atypically up-regulated during endodormancy included those encoding transcription factors associated with responses to cold and other abiotic stresses (*ZAT10/STZ, ZAT12/RHL41, WRKY*), and a gene that seems to encode a trihelix transcription factor. The down-regulation of other transcription factor genes was consistent with changes known to accompany endodormancy. These include genes with various roles in auxin signaling, organization of the shoot apical meristem, and organ development.

We identified many novel and previously identified promoter motifs that appear to regulate these dormancy-associated changes in gene expression. The most common motifs were those associated with the circadian clock and others associated with responses to photoperiod, cold, dehydration, and ABA. Among the most common motifs were the EVENING ELEMENT-LIKE motif, a binding site found in genes targeted by CCA1, CBF-binding sites, and various ABA responsive elements.

Finally, we found many differentially expressed genes that were located near bud set QTL, some of which are clear candidates for having functional roles in the induction of endodormancy. These latter genes are potential targets for basic research and for manipulating dormancy-associated processes using molecular breeding and transgenic approaches. Additional gene expression, fine-scale mapping, functional, and population genetic studies should help elucidate the roles of the many genes and biological processes we identified.

## Conflict of Interest Statement

The authors declare that the research was conducted in the absence of any commercial or financial relationships that could be construed as a potential conflict of interest.

## Footnotes

1 http://www.nimblegen.com/

2 http://www.phytozome.net/

3 http://ncbi.nlm.nih.gov/geo/

4 http://www.dna.affrc.go.jp/PLACE/

5 http://bioinformatics.psb.ugent.be/webtools/plantcare/html/

6 ftp://ftp.arabidopsis.org/Genes/gene_aliases_20130831.txt

7 http://planttfdb.cbi.pku.edu.cn/

8 http://www.broadinstitute.org/gsea/index.jsp

9 http://geneontology.org/

10 http://www.phytozome.net/

## Supplementary material

FIGURE S1 **Average monthly temperature and precipitation in Corvallis, OR, USA during the sample collection period**.

FIGURE S2 **Bud morphology and histology during the sample collection period**.
