# Supplementary material for: Extensive Transcriptome Changes During Natural Onset and Release of Vegetative Bud Dormancy in Populus
Source: Front Plant Sci. 2015 Dec 17;6:989. doi: 10.3389/fpls.2015.00989 (PMC4681841; doi:10.3389/fpls.2015.00989)
Supplement: FIGURE S1 — Average monthly temperature and precipitation in Corvallis, OR, USA during the sample collection period. [file Figures_S1-S2.PDF]

Supplementary Material

Extensive transcriptome changes during natural onset and release of vegetative bud dormancy in *Populus*

Glenn T. Howe[1†], David P. Horvath[2†], Palitha Dharmawardhana[1,3], Henry D. Priest[4,5], Todd C. Mockler[3,4], and Steven H. Strauss[1]*

[1]Department of Forest Ecosystems and Society, Oregon State University, Corvallis, OR, USA, [2]Biosciences Research Laboratory, USDA-Agricultural Research Service, Fargo, ND, USA, [3]Department of Botany and Plant Pathology, Oregon State University, Corvallis, OR, USA, [4]Donald Danforth Plant Science Center, Saint Louis, MO, USA, [5]Division of Biology and Biomedical Sciences, Washington University, Saint Louis, MO, USA

[†]These authors contributed equally to his work.

***Correspondence:** Steven H. Strauss, Department of Forest Ecosystems and Society, Oregon State University, 321 Richardson Hall, Corvallis, OR, 97331-5752, USA.
Steve.Strauss@oregonstate.edu

1. Supplementary Figures

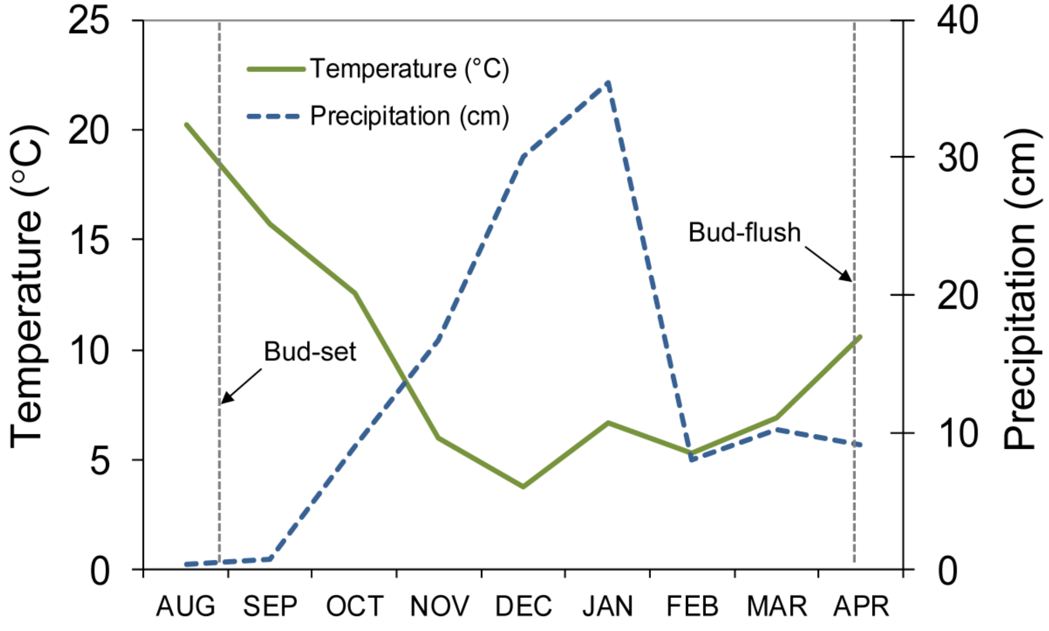

Figure S1. Average monthly temperature and precipitation in Corvallis OR during the sample collection period.

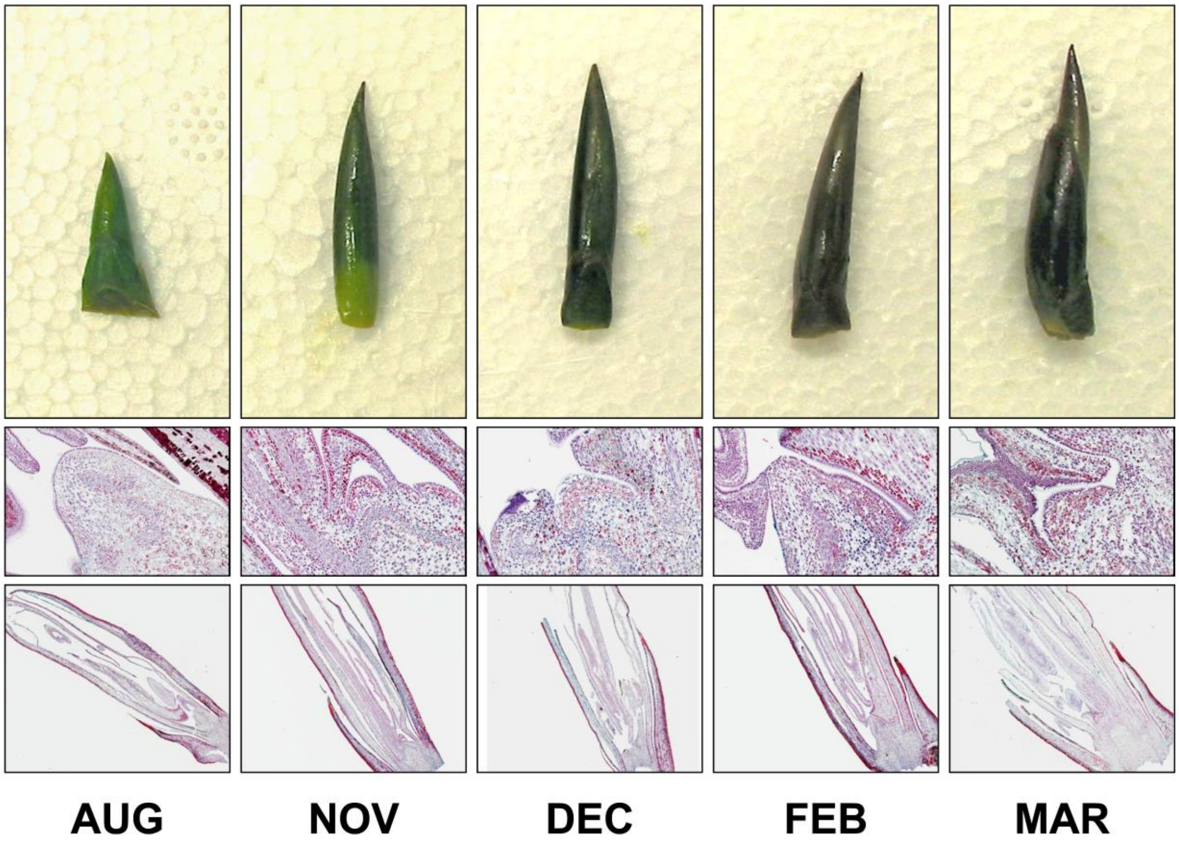

Figure S2. Bud morphology and histology during the sample collection period.
