# Supplementary material for: Extensive Transcriptome Changes During Natural Onset and Release of Vegetative Bud Dormancy in Populus
Source: Front Plant Sci. 2015 Dec 17;6:989. doi: 10.3389/fpls.2015.00989 (PMC4681841; doi:10.3389/fpls.2015.00989)
Supplement: Supplementary file 2 [file Tables_S1-S8.PDF]

Table S1. Gene ontology (GO) biological process gene sets differentially expressed between paradormancy and endodormancy. 'Up-regulated' and 'down-regulated' gene sets are enriched for genes that either increased or decreased in gene expression, respectively. 'Up- or down-regulated' gene sets are enriched for genes that changed in gene expression, ignoring the direction of change of the individual genes. For the 'up- or down-regulated' gene sets, '**' denotes that the gene set was not significant in the other two analyses, whereas '+' and '-' indicate that the gene set was also enriched for genes that were up-regulated or down-regulated, respectively. Gene sets that were significant at an FDR q-value < 0.10 are identified by their GO terms and corresponding GO identifiers (in parentheses). FDR-U, FDR-D, and FDR-E are FDR q-values for up-regulated, down-regulated, and up- or down-regulated gene sets.

**Up-regulated (U)**	**FDR-U**	**Down-regulated (D)**	**FDR-D**	**Up- or down-regulated (E)**	**FDR-E**
Galactose metabolic process (0006012)	0.000	GTP catabolic process (0006184)	0.000	Photosynthesis, light harvesting (0009765) (-)	0.000
Apoptotic process (0006915)	0.052	DNA replication (0006260)	0.000	Isoprenoid biosynthetic process (0008299) (-)	0.001
Recognition of pollen (0048544)	0.063	Base-excision repair (0006284)	0.000	Lipid biosynthetic process (0008610) (-)	0.003
Cell wall macromolecule catabolic process (0016998)	0.070	Nucleosome assembly (0006334)	0.000	Galactose metabolic process (0006012) (+)	0.004
Response to wounding (0009611)	0.081	Translational elongation (0006414)	0.000	Glutamine biosynthetic process (0006542) (-)	0.008
Chitin catabolic process (0006032)	0.086	Protein folding (0006457)	0.000	Protein polymerization (0051258) (-)	0.014
Protein insertion into membrane (0051205)	0.089	Microtubule-based process (0007017)	0.000	Fatty acid biosynthetic process (0006633) (-)	0.016
Guanosine tetraphosphate metabolic process (0015969)	0.098	Microtubule-based movement (0007018)	0.000	Microtubule-based process (0007017) (-)	0.022
		Lipid biosynthetic process (0008610)	0.000	L-phenylalanine biosynthetic process (0009094) (-)	0.024
		Cellular amino acid biosynthetic process (0008652)	0.000	GTP catabolic process (0006184) (-)	0.025
		Aromatic amino acid family biosynthetic process (0009073)	0.000	Nucleosome assembly (0006334) (-)	0.026
		Photosynthesis, light harvesting (0009765)	0.000	Aromatic amino acid family biosynthetic process (0009073) (-)	0.027
		Protein polymerization (0051258)	0.000	Signal peptide processing (0006465) (-)	0.034
		Proteolysis involved in cellular protein catabolic process (0051603)	0.000	Cellular amino acid biosynthetic process (0008652) (-)	0.037
		Signal peptide processing (0006465)	0.000	Cellular amino acid metabolic process (0006520)**	0.058
		Isoprenoid biosynthetic process (0008299)	0.000	Chlorophyll catabolic process (0015996) (-)	0.061
		ATP synthesis coupled proton transport (0015986)	0.001	Phosphatidylinositol phosphorylation (0046854)**	0.063
		Cellular protein metabolic process (0044267)	0.004	Carbon utilization (0015976)**	0.063
		Fatty acid biosynthetic process (0006633)	0.005	Photosynthesis (0015979) (-)	0.063
		Terpenoid biosynthetic process (0016114)	0.005	Cellular aldehyde metabolic process (0006081)**	0.064

Table S1. Gene ontology (GO) biological process gene sets differentially expressed between paradormancy and endodormancy. 'Up-regulated' and 'down-regulated' gene sets are enriched for genes that either increased or decreased in gene expression, respectively. 'Up- or down-regulated' gene sets are enriched for genes that changed in gene expression, ignoring the direction of change of the individual genes. For the 'up- or down-regulated' gene sets, '**' denotes that the gene set was not significant in the other two analyses, whereas '+' and '-' indicate that the gene set was also enriched for genes that were up-regulated or down-regulated, respectively. Gene sets that were significant at an FDR q-value < 0.10 are identified by their GO terms and corresponding GO identifiers (in parentheses). FDR-U, FDR-D, and FDR-E are FDR q-values for up-regulated, down-regulated, and up- or down-regulated gene sets.

Up-regulated (U)	FDR-U	Down-regulated (D)	FDR-D	Up- or down-regulated (E)	FDR-E
		Protein import into mitochondrial inner membrane (0045039)	0.006	Translational elongation (0006414) (-)	0.064
		Protein targeting to mitochondrion (0006626)	0.008	L-serine biosynthetic process (0006564)**	0.064
		Steroid biosynthetic process (0006694)	0.010	CTP biosynthetic process (0006241) (-)	0.069
		Photosynthesis (0015979)	0.011	Phosphatidylinositol metabolic process (0046488)**	0.070
		Protein methylation (0006479)	0.011	Polysaccharide catabolic process (0000272)**	0.072
		Histidine biosynthetic process (0000105)	0.011	Cytoskeleton organization (0007010) (-)	0.073
		Small GTPase mediated signal transduction (0007264)	0.011	Protein import into mitochondrial inner membrane (0045039) (-)	0.073
		Response to hormone stimulus (0009725)	0.012	Protein targeting to mitochondrion (0006626) (-)	0.074
		Glutamine biosynthetic process (0006542)	0.012	Nitrogen compound metabolic process (0006807) (-)	0.075
		Ribosome biogenesis (0042254)	0.012	GTP biosynthetic process (0006183) (-)	0.075
		Isocitrate metabolic process (0006102)	0.012	Nucleoside diphosphate phosphorylation (0006165) (-)	0.075
		ATP hydrolysis coupled proton transport (0015991)	0.013	Terpenoid biosynthetic process (0016114) (-)	0.076
		Chlorophyll biosynthetic process (0015995)	0.016	UTP biosynthetic process (0006228) (-)	0.077
		Cytoskeleton organization (0007010)	0.025	Cell cycle (0007049) (-)	0.089
		Nucleoside diphosphate phosphorylation (0006165)	0.029	tRNA aminoacylation (0043039)**	0.094
		Cell cycle (0007049)	0.033	Carboxylic acid metabolic process (0019752)**	0.096
		GTP biosynthetic process (0006183)	0.033	Branched-chain amino acid metabolic process (0009081)**	0.097
		CTP biosynthetic process (0006241)	0.034	Transcription initiation from RNA pol II promoter (0006367)**	0.099
		Cellular metabolic process (0044237)	0.034	ER to golgi vesicle-mediated transport (0006888)**	0.099
		rRNA modification (0000154)	0.046		
		UTP biosynthetic process (0006228)	0.048		

Table S1. Gene ontology (GO) biological process gene sets differentially expressed between paradormancy and endodormancy. 'Up-regulated' and 'down-regulated' gene sets are enriched for genes that either increased or decreased in gene expression, respectively. 'Up- or down-regulated' gene sets are enriched for genes that changed in gene expression, ignoring the direction of change of the individual genes. For the 'up- or down-regulated' gene sets, '**' denotes that the gene set was not significant in the other two analyses, whereas '+' and '-' indicate that the gene set was also enriched for genes that were up-regulated or down-regulated, respectively. Gene sets that were significant at an FDR q-value < 0.10 are identified by their GO terms and corresponding GO identifiers (in parentheses). FDR-U, FDR-D, and FDR-E are FDR q-values for up-regulated, down-regulated, and up- or down-regulated gene sets.

Up-regulated (U)	FDR-U	Down-regulated (D)	FDR-D	Up- or down-regulated (E)	FDR-E
		Mitochondrial electron transport, ubiquinol to cytochrome c (0006122)	0.048		
		Chlorophyll catabolic process (0015996)	0.048		
		Nitrogen compound metabolic process (0006807)	0.056		
		Glycolysis (0006096)	0.059		
		L-phenylalanine biosynthetic process (0009094)	0.064		
		Cell redox homeostasis (0045454)	0.072		
		Cell cycle arrest (0007050)	0.082		
		Cell division (0051301)	0.086		
		Pentose-phosphate shunt (0006098)	0.100		

Table S2. Gene ontology (GO) biological process gene sets differentially expressed between endodormancy and ecodormancy. 'Up-regulated' and 'down-regulated' gene sets are enriched for genes that either increased or decreased in gene expression, respectively. 'Up- or down-regulated' gene sets are enriched for genes that changed in gene expression, ignoring the direction of change of the individual genes. For the 'up- or down-regulated' gene sets, '**' denotes that the gene set was not significant in the other two analyses, whereas '+' and '-' indicate that the gene set was also enriched for genes that were up-regulated or down-regulated, respectively. Gene sets that were significant at an FDR q-value < 0.10 are identified by their GO terms and corresponding GO identifiers (in parentheses). FDR-U, FDR-D, and FDR-E are FDR q-values for up-regulated, down-regulated, and up- or down-regulated gene sets.

**Up-regulated (U)**	**FDR-U**	**Down-regulated (D)**	**FDR-D**	**Up- or down-regulated (E)**	**FDR-E**
Negative regulation of catalytic activity (0043086)	0.000	Galactose metabolic process (0006012)	0.000	Polysaccharide catabolic process (0000272)**	0.096
Base-excision repair (0006284)	0.004	Photosynthetic electron transport chain (0009767)	0.000	Protein import into mitochondrial inner membrane (0045039) (+)	0.099
Microtubule-based movement (0007018)	0.005	ATP hydrolysis coupled proton transport (0015991)	0.000		
DNA replication (0006260)	0.005	Photosynthesis, light reaction (0019684)	0.000		
Cellular protein metabolic process (0044267)	0.007	Protein transport (0015031)	0.005		
Protein polymerization (0051258)	0.009	Autophagic vacuole assembly (0000045)	0.010		
Translational elongation (0006414)	0.010	Response to water stimulus (0009415)	0.024		
Photosynthesis, light harvesting (0009765)	0.011	Lipopolysaccharide biosynthetic process (0009103)	0.027		
Protein targeting to mitochondrion (0006626)	0.015	Respiratory electron transport chain (0022904)	0.030		
Protein import into mitochondrial inner membrane (0045039)	0.019	ATP metabolic process (0046034)	0.077		
GTP catabolic process (0006184)	0.020	Carbon fixation (0015977)	0.078		
Ribosome biogenesis (0042254)	0.034	RNA polyadenylation (0043631)	0.079		
Terpenoid biosynthetic process (0016114)	0.041	ATP synthesis coupled electron transport (0042773)	0.082		
Response to light stimulus (0009416)	0.042	Response to wounding (0009611)	0.087		
Microtubule-based process (0007017)	0.045	Golgi vesicle transport (0048193)	0.091		
Amine metabolic process (0009308)	0.049				
Protein folding (0006457)	0.051				
rRNA processing (0006364)	0.055				

Table S3. Gene ontology (GO) molecular function gene sets differentially expressed between paradormancy and endodormancy. 'Up-regulated' and 'down-regulated' gene sets are enriched for genes that either increased or decreased in gene expression, respectively. 'Up- or down-regulated' gene sets are enriched for genes that changed in gene expression, ignoring the direction of change of the individual genes. For the 'up- or down-regulated' gene sets, '**' denotes that the gene set was not significant in the other two analyses, whereas '+' and '-' indicate that the gene set was also enriched for genes that were up-regulated or down-regulated, respectively. Gene sets that were significant at an FDR q-value < 0.10 are identified by their GO terms and corresponding GO identifiers (in parentheses). FDR-U, FDR-D, and FDR-E are FDR q-values for up-regulated, down-regulated, and up- or down-regulated gene sets.

**Up-regulated (U)**	**FDR-U**	**Down-regulated (D)**	**FDR-D**	**Up- or down-regulated (E)**	**FDR-E**
UDP-glucose 4-epimerase activity (0003978)	0.008	Structural constituent of ribosome (0003735)	0.000	Ammonia-lyase activity (0016841) (-)	0.001
Ionotropic glutamate receptor activity (0004970)	0.009	Microtubule motor activity (0003777)	0.000	Glutamate-ammonia ligase activity (0004356) (-)	0.001
Extracellular-glutamate-gated ion channel activity (0005234)	0.012	Threonine-type endopeptidase activity (0004298)	0.000	Structural constituent of ribosome (0003735) (-)	0.002
ADP binding (0043531)	0.021	rRNA binding (0019843)	0.000	Catechol oxidase activity (0004097) (-)	0.006
Squalene monooxygenase activity (0004506)	0.030	Endopeptidase activity (0004175)	0.000	UDP-glucose 4-epimerase activity (0003978) (+)	0.020
Starch binding (2001070)	0.033	Cytochrome-c oxidase activity (0004129)	0.000	Antioxidant activity (0016209) (-)	0.022
Binding (0005488)	0.050	Ammonia-lyase activity (0016841)	0.000	Homocysteine S-methyltransferase activity (0008898)**	0.028
Ligase activity, forming aminoacyl-tRNA and related compounds (0016876)	0.056	GTPase activity (0003924)	0.000	2 iron, 2 sulfur cluster binding (0051537)**	0.038
Sigma factor activity (0016987)	0.064	Unfolded protein binding (0051082)	0.001	Ketol-acid reductoisomerase activity (0004455) (-)	0.038
UDP-n-acetylmuramate dehydrogenase activity (0008762)	0.077	Catechol oxidase activity (0004097)	0.002	Glutathione peroxidase activity (0004602)**	0.039
Glycolipid transporter activity (0017089)	0.079	tRNA binding (0000049)	0.002	Shikimate 3-dehydrogenase (NADP+) activity (0004764) (-)	0.039
Phosphatidylinositol phospholipase c activity (0004435)	0.081	GTP binding (0005525)	0.004	Prephenate dehydratase activity (0004664) (-)	0.040
Alpha-amylase activity (0004556)	0.083	Oxidoreductase activity, acting on the CH-OH group of donors, NAD or NADP as acceptor (0016616)	0.004	Transferase activity, transferring acyl groups (0016746) (-)	0.041
Transferase activity, transferring glycosyl groups (0016757)	0.084	RNA-directed RNA polymerase activity (0003968)	0.004	3-oxoacyl-[acyl-carrier-protein] synthase activity (0004315)**	0.042
Chitinase activity (0004568)	0.085	NAD binding (0051287)	0.004	Actin binding (0003779) (-)	0.042
Glycolipid binding (0051861)	0.086	Actin binding (0003779)	0.004	Chlorophyllase activity (0047746) (-)	0.059
Citrate transmembrane transporter activity (0015137)	0.087	Translation elongation factor activity (0003746)	0.004	Binding (0005488) (+)	0.075
Cation transmembrane transporter activity (0008324)	0.088	Oxidoreductase activity, acting on single donors with incorporation of molecular oxygen, incorporati	0.007	Cofactor binding (0048037) (-)	0.077

Table S3. Gene ontology (GO) molecular function gene sets differentially expressed between paradormancy and endodormancy. 'Up-regulated' and 'down-regulated' gene sets are enriched for genes that either increased or decreased in gene expression, respectively. 'Up- or down-regulated' gene sets are enriched for genes that changed in gene expression, ignoring the direction of change of the individual genes. For the 'up- or down-regulated' gene sets, '**' denotes that the gene set was not significant in the other two analyses, whereas '+' and '-' indicate that the gene set was also enriched for genes that were up-regulated or down-regulated, respectively. Gene sets that were significant at an FDR q-value < 0.10 are identified by their GO terms and corresponding GO identifiers (in parentheses). FDR-U, FDR-D, and FDR-E are FDR q-values for up-regulated, down-regulated, and up- or down-regulated gene sets.

**Up-regulated (U)**	**FDR-U**	**Down-regulated (D)**	**FDR-D**	**Up- or down-regulated (E)**	**FDR-E**
Serine-type endopeptidase inhibitor activity (0004867)	0.090	Cofactor binding (0048037)	0.007	Oxidoreductase activity, acting on CH-OH group of donors (0016614)**	0.077
Acyl-CoA dehydrogenase activity (0003995)	0.091	Transferase activity, transferring acyl groups (0016746)	0.008	Ribonucleoside-diphosphate reductase activity, thioredoxin disulfide as acceptor (0004748)**	0.080
		RNA binding (0003723)	0.011	Carbonate dehydratase activity (0004089)**	0.082
		Rho guanyl-nucleotide exchange factor activity (0005089)	0.011	Protein domain specific binding (0019904) (-)	0.084
		Coenzyme binding (0050662)	0.012	Phosphoglycerate dehydrogenase activity (0004617)**	0.084
		Transferase activity, transferring acyl groups other than amino-acyl groups (0016747)	0.013	Aldehyde dehydrogenase [NAD(p)+] activity (0004030)**	0.085
		Peptidase activity (0008233)	0.013	rRNA binding (0019843) (-)	0.085
		Protein methyltransferase activity (0008276)	0.014	Cytochrome-c oxidase activity (0004129) (-)	0.086
		Chaperone binding (0051087)	0.017	Alpha-amylase activity (0004556) (+)	0.095
		Isocitrate dehydrogenase (NADP+) activity (0004450)	0.017		
		3-deoxy-7-phosphoheptulonate synthase activity (0003849)	0.024		
		Hydrogen ion transporting ATP synthase activity, rotational mechanism (0046933)	0.027		
		Antioxidant activity (0016209)	0.027		
		3-beta-hydroxy-delta5-steroid dehydrogenase activity (0003854)	0.027		
		Nucleoside diphosphate kinase activity (0004550)	0.037		
		5S rRNA binding (0008097)	0.042		
		Protein domain specific binding (0019904)	0.043		
		rRNA methyltransferase activity (0008649)	0.043		
		Beta-galactosidase activity (0004565)	0.044		

Table S3. Gene ontology (GO) molecular function gene sets differentially expressed between paradormancy and endodormancy. ‘Up-regulated’ and ‘down-regulated’ gene sets are enriched for genes that either increased or decreased in gene expression, respectively. ‘Up- or down-regulated’ gene sets are enriched for genes that changed in gene expression, ignoring the direction of change of the individual genes. For the ‘up- or down-regulated’ gene sets, ‘**’ denotes that the gene set was not significant in the other two analyses, whereas ‘+’ and ‘-’ indicate that the gene set was also enriched for genes that were up-regulated or down-regulated, respectively. Gene sets that were significant at an FDR q-value < 0.10 are identified by their GO terms and corresponding GO identifiers (in parentheses). FDR-U, FDR-D, and FDR-E are FDR q-values for up-regulated, down-regulated, and up- or down-regulated gene sets.

Up-regulated (U)	FDR-U	Down-regulated (D)	FDR-D	Up- or down-regulated (E)	FDR-E
		Proton-transporting ATPase activity, rotational mechanism (0046961)	0.046		
		Hydrogen ion transmembrane transporter activity (0015078)	0.046		
		Glutamate-ammonia ligase activity (0004356)	0.048		
		Aspartic-type endopeptidase activity (0004190)	0.049		
		Triose-phosphate isomerase activity (0004807)	0.051		
		Oxidoreductase activity, acting on the aldehyde or oxo group of donors, NAD or NADP as acceptor (001	0.052		
		Chlorophyllase activity (0047746)	0.054		
		Structural molecule activity (0005198)	0.054		
		Shikimate 3-dehydrogenase (NADP+) activity (0004764)	0.060		
		rRNA (adenine-n6,n6-)-dimethyltransferase activity (0000179)	0.060		
		DNA-3-methyladenine glycosylase activity (0008725)	0.061		
		Magnesium chelatase activity (0016851)	0.061		
		Methylmalonate-semialdehyde dehydrogenase (acylating) activity (0004491)	0.066		
		Prenyltransferase activity (0004659)	0.066		
		Ketol-acid reductoisomerase activity (0004455)	0.073		
		3-dehydroquinate dehydratase activity (0003855)	0.087		
		Asparagine synthase (glutamine-hydrolyzing) activity (0004066)	0.088		
		Hydrolase activity, acting on ester bonds (0016788)	0.088		

Table S3. Gene ontology (GO) molecular function gene sets differentially expressed between paradormancy and endodormancy. 'Up-regulated' and 'down-regulated' gene sets are enriched for genes that either increased or decreased in gene expression, respectively. 'Up- or down-regulated' gene sets are enriched for genes that changed in gene expression, ignoring the direction of change of the individual genes. For the 'up- or down-regulated' gene sets, '**' denotes that the gene set was not significant in the other two analyses, whereas '+' and '-' indicate that the gene set was also enriched for genes that were up-regulated or down-regulated, respectively. Gene sets that were significant at an FDR q-value < 0.10 are identified by their GO terms and corresponding GO identifiers (in parentheses). FDR-U, FDR-D, and FDR-E are FDR q-values for up-regulated, down-regulated, and up- or down-regulated gene sets.

Up-regulated (U)	FDR-U	Down-regulated (D)	FDR-D	Up- or down-regulated (E)	FDR-E
		Nucleobase-containing compound kinase activity (0019205)	0.089		
		Histone-lysine n-methyltransferase activity (0018024)	0.090		
		Fructose-bisphosphate aldolase activity (0004332)	0.093		
		Ubiquinol-cytochrome-c reductase activity (0008121)	0.093		
		Phosphogluconate dehydrogenase (decarboxylating) activity (0004616)	0.094		
		Prephenate dehydratase activity (0004664)	0.094		
		Bile acid (0008508)	0.094		
		Magnesium ion binding (0000287)	0.095		
		Hydrolase activity (0016787)	0.096		
		Ribonuclease III activity (0004525)	0.096		
		DNA helicase activity (0003678)	0.097		

Table S4. Gene ontology (GO) molecular function gene sets differentially expressed between endodormancy and ecodormancy. 'Up-regulated' and 'down-regulated' gene sets are enriched for genes that either increased or decreased in gene expression, respectively. 'Up- or down-regulated' gene sets are enriched for genes that changed in gene expression, ignoring the direction of change of the individual genes. For the 'up- or down-regulated' gene sets, '**' denotes that the gene set was not significant in the other two analyses, whereas '+' and '-' indicate that the gene set was also enriched for genes that were up-regulated or down-regulated, respectively. Gene sets that were significant at an FDR q-value < 0.10 are identified by their GO terms and corresponding GO identifiers (in parentheses). FDR-U, FDR-D, and FDR-E are FDR q-values for up-regulated, down-regulated, and up- or down-regulated gene sets.

Up-regulated (U)	FDR-U	Down-regulated (D)	FDR-D	Up- or down-regulated (E)	FDR-E
Structural constituent of ribosome (0003735)	0.000	Chlorophyll binding (0016168)	0.001	Beta-amylase activity (0016161)**	0.095
Identical protein binding (0042802)	0.000	Acid-amino acid ligase activity (0016881)	0.036		
Nutrient reservoir activity (0045735)	0.005	UDP-glucose 4-epimerase activity (0003978)	0.036		
Rho guanyl-nucleotide exchange factor activity (0005089)	0.006	Translation release factor activity, codon specific (0016149)	0.041		
Microtubule motor activity (0003777)	0.006	Phosphotransferase activity, alcohol group as acceptor (0016773)	0.042		
DNA-3-methyladenine glycosylase activity (0008725)	0.022	Hydrolase activity, acting on acid anhydrides, catalyzing transmembrane movement of substances (0016820)	0.052		
Ribonucleoside-diphosphate reductase activity, thioredoxin disulfide as acceptor (0004748)	0.076	Cysteine-type endopeptidase inhibitor activity (0004869)	0.055		
Hydrolase activity, acting on ester bonds (0016788)	0.082	Serine-type endopeptidase inhibitor activity (0004867)	0.080		
Sulfotransferase activity (0008146)	0.084	Phosphotransferase activity, for other substituted phosphate groups (0016780)	0.083		
		Glutathione peroxidase activity (0004602)	0.088		
		Chitin binding (0008061)	0.096		

Table S5. Pathway Studio gene sets and subnetworks differentially expressed between paradormancy and endodormancy. 'Up-regulated' and 'down-regulated' gene sets are enriched for genes that either increased or decreased in gene expression, respectively. 'Up- or down-regulated' gene sets are enriched for genes that changed in gene expression, ignoring the direction of change of the individual genes. For the 'up- or down-regulated' gene sets, '**' denotes that the gene set was not significant in the other two analyses, whereas '+' and '-' indicate that the gene set was also enriched for genes that were up-regulated or down-regulated, respectively. Gene sets that were significant at an FDR q-value < 0.10 are identified by their Pathway Studio terms. FDR-U, FDR-D, and FDR-E are FDR q-values for up-regulated, down-regulated, and up- or down-regulated gene sets.

Up-regulated (U)	FDR-U	Down-regulated (D)	FDR-D	Up- or down-regulated (E)	FDR-E
Expression targets of WRKY	0.034	Expression targets of TT2	0.000	Neighbors of PAP1 (-)	0.003
Neighbors of laminaran	0.040	Neighbors of AS1	0.000	Expression targets of TT2 (-)	0.003
Neighbors of ZTL	0.042	Neighbors of MYB90	0.000	Neighbors of TT2 (-)	0.004
Neighbors of RHL41	0.044	Neighbors of PAP1	0.000	Regulators of proanthocyanidin metabolism (-)	0.004
Regulators of photoperiodism; flowering	0.044	Neighbors of TT2	0.000	Neighbors of MYB90 (-)	0.005
Expression targets of ACD6	0.047	Regulators of organ formation	0.000	Neighbors of cGMP (-)	0.006
Regulators of cellularization of megagametophyte	0.047	Binding partners of ISP	0.000	Expression targets of HY5 (-)	0.006
Neighbors of FIE	0.048	Regulators of plastid fission	0.000	Regulators of response to cold (-)	0.007
Neighbors of BZIP transcription factor	0.048	Regulators of meristem growth	0.000	Neighbors of SAD2 (-)	0.007
Neighbors of spermine	0.049	Regulators of cuticle development	0.000	Binding partners of CP47 (-)	0.007
Neighbors of NPR3	0.049	Regulators of microtubule cytoskeleton assembly	0.000	Regulators of plant stress**	0.007
Expression targets of SFR6	0.049	Regulators of proanthocyanidin metabolism	0.000	Neighbors of floraltone (-)	0.008
Expression targets of ZTL	0.050	Neighbors of MYB-factors	0.000	Neighbors of ABCB19 (-)	0.008
Neighbors of WRKY33	0.053	Neighbors of GL3	0.000	Neighbors of beta-aminobutyric acid (-)	0.008
Neighbors of ACD6	0.055	Neighbors of SAD2	0.000	Neighbors of MIR156A (-)	0.008
Neighbors of NDR1	0.059	Regulators of chloroplast division	0.000	Neighbors of deacetylchitin (-)	0.008
Regulators of daylength response	0.059	Regulators of flower patterning	0.000	Binding partners of GL1 (-)	0.008
Neighbors of SAL1	0.059	Neighbors of HSF	0.000	Regulators of tricarboxylic acid cycle**	0.008
Expression targets of LWD2	0.067	Neighbors of AS2	0.001	Binding partners of ISP (-)	0.008
Neighbors of SFR6	0.068	Regulators of leaf patterning	0.001	Neighbors of ABF2 (-)	0.008
Neighbors of EIN2	0.070	Neighbors of N-benzyladenine	0.001	Binding partners of PRL1 (-)	0.008
Neighbors of LWD2	0.071	Regulators of cell cycle	0.001	Targets of MIR156A (-)	0.008
Regulators of exocytosis	0.076	Neighbors of calcosan	0.001	Regulators of cuticle development (-)	0.008

Table S5. Pathway Studio gene sets and subnetworks differentially expressed between paradormancy and endodormancy. 'Up-regulated' and 'down-regulated' gene sets are enriched for genes that either increased or decreased in gene expression, respectively. 'Up- or down-regulated' gene sets are enriched for genes that changed in gene expression, ignoring the direction of change of the individual genes. For the 'up- or down-regulated' gene sets, '**' denotes that the gene set was not significant in the other two analyses, whereas '+' and '-' indicate that the gene set was also enriched for genes that were up-regulated or down-regulated, respectively. Gene sets that were significant at an FDR q-value < 0.10 are identified by their Pathway Studio terms. FDR-U, FDR-D, and FDR-E are FDR q-values for up-regulated, down-regulated, and up- or down-regulated gene sets.

Up-regulated (U)	FDR-U	Down-regulated (D)	FDR-D	Up- or down-regulated (E)	FDR-E
Neighbors of ARF6	0.077	Neighbors of ABCB19	0.001	Expression targets of SHB1 (-)	0.008
Regulators of chloroplast function	0.078	Neighbors of TTG1	0.001	Neighbors of CPR5**	0.009
Neighbors of WRKY	0.079	Neighbors of cGMP	0.001	Regulators of plant infection (-)	0.010
Expression targets of MEK1	0.079	Neighbors of SEU	0.001	Expression targets of AVRRPM1**	0.012
Neighbors of APX1	0.080	Neighbors of JLO	0.002	Neighbors of SHB1 (-)	0.013
Neighbors of AVRPTO	0.081	Regulators of response to cold	0.002	Targets of MIR393A (-)	0.015
Neighbors of EIN3	0.082	Regulators of flavonol metabolism	0.002	Targets of MIR156G (-)	0.015
Neighbors of VTC2	0.084	Regulators of pistil development	0.002	Targets of MIR156C (-)	0.017
Expression targets of endo-1,4-beta-xylanase	0.088	Regulators of response to gravity	0.002	Targets of MIR156E (-)	0.017
Neighbors of PBS2	0.088	Expression targets of AS2	0.003	Neighbors of MIR156C (-)	0.017
Neighbors of carotenoids	0.088	Neighbors of geldanamycin	0.003	Targets of MIR156F (-)	0.017
Expression targets of EIN3	0.089	Neighbors of RDR6	0.004	Regulators of organ formation (-)	0.017
Regulators of endosperm cellularization	0.089	Neighbors of FHY1	0.004	Neighbors of MIR156E (-)	0.017
Neighbors of SOS1	0.089	Binding partners of EGL3	0.005	Regulators of response to gravity (-)	0.017
Expression targets of ENO1	0.090	Neighbors of steroids	0.005	Targets of MIR156B (-)	0.017
Neighbors of chlorethephon	0.090	Regulators of meristem organization	0.005	Neighbors of MIR156G (-)	0.017
Neighbors of ICE1	0.092	Expression targets of SHB1	0.006	Neighbors of MIR393A (-)	0.018
Neighbors of ENO1	0.092	Targets of MIR393A	0.006	Binding partners of PSAH-1 (-)	0.018
Neighbors of ATAN11	0.094	Neighbors of MIR393A	0.007	Expression targets of ETR1**	0.018
Neighbors of NRT1.1	0.094	Neighbors of SHB1	0.007	Neighbors of diuron**	0.018
Expression targets of ABA2	0.096	Neighbors of benzoadenosine	0.007	Regulators of DNA damage (-)	0.018
		Regulators of cotyledon development	0.007	Targets of MIR156D (-)	0.018
		Neighbors of BOP1	0.007	Binding partners of PSAK**	0.018

Table S5. Pathway Studio gene sets and subnetworks differentially expressed between paradormancy and endodormancy. 'Up-regulated' and 'down-regulated' gene sets are enriched for genes that either increased or decreased in gene expression, respectively. 'Up- or down-regulated' gene sets are enriched for genes that changed in gene expression, ignoring the direction of change of the individual genes. For the 'up- or down-regulated' gene sets, '**' denotes that the gene set was not significant in the other two analyses, whereas '+' and '-' indicate that the gene set was also enriched for genes that were up-regulated or down-regulated, respectively. Gene sets that were significant at an FDR q-value < 0.10 are identified by their Pathway Studio terms. FDR-U, FDR-D, and FDR-E are FDR q-values for up-regulated, down-regulated, and up- or down-regulated gene sets.

Up-regulated (U)	FDR-U	Down-regulated (D)	FDR-D	Up- or down-regulated (E)	FDR-E
		Targets of MIR156A	0.007	Neighbors of MIR156D (-)	0.019
		Binding partners of PRL1	0.007	Neighbors of HY5**	0.021
		Regulators of G2/M transition	0.007	Neighbors of SOS2**	0.022
		Expression targets of calmodulin	0.007	Regulators of induced systemic resistance**	0.023
		Regulators of DNA damage	0.007	Neighbors of N-benzyladenine (-)	0.024
		Neighbors of LUG	0.007	Neighbors of MIR157D (-)	0.024
		Expression targets of GBF3	0.007	Expression targets of ENO1 (+)	0.024
		Regulators of spindle assembly	0.007	Neighbors of MIR156B (-)	0.024
		Regulators of post-transcriptional gene silencing	0.007	Neighbors of HYH (-)	0.025
		Neighbors of SWN	0.007	Targets of MIR157D (-)	0.026
		Neighbors of L-serine	0.007	Neighbors of PDS3**	0.027
		Neighbors of heat shock	0.007	Expression targets of JAR1**	0.027
		Neighbors of HSFA2	0.007	Neighbors of FHY1 (-)	0.027
		Expression targets of MYB-factors	0.008	Regulators of cotyledon development (-)	0.027
		Neighbors of CMT3	0.008	Neighbors of ENO1 (+)	0.030
		Neighbors of MIR156A	0.008	Neighbors of CPC (-)	0.030
		Neighbors of GBF3	0.008	Neighbors of GL3 (-)	0.030
		Regulators of brassinosteroid mediated signaling	0.008	Neighbors of RHL41 (+)	0.030
		Binding partners of PSAH-1	0.010	Regulators of interphase**	0.031
		Regulators of response to DNA damage	0.010	Neighbors of L-glutamate**	0.033
		Regulators of fatty acid elongation	0.010	Neighbors of SEU (-)	0.034
		Regulators of phenylpropanoid metabolism	0.010	Neighbors of G-protein (-)	0.034
		Expression targets of JLO	0.010	Neighbors of L-glutamine (-)	0.034

Table S5. Pathway Studio gene sets and subnetworks differentially expressed between paradormancy and endodormancy. 'Up-regulated' and 'down-regulated' gene sets are enriched for genes that either increased or decreased in gene expression, respectively. 'Up- or down-regulated' gene sets are enriched for genes that changed in gene expression, ignoring the direction of change of the individual genes. For the 'up- or down-regulated' gene sets, '**' denotes that the gene set was not significant in the other two analyses, whereas '+' and '-' indicate that the gene set was also enriched for genes that were up-regulated or down-regulated, respectively. Gene sets that were significant at an FDR q-value < 0.10 are identified by their Pathway Studio terms. FDR-U, FDR-D, and FDR-E are FDR q-values for up-regulated, down-regulated, and up- or down-regulated gene sets.

Up-regulated (U)	FDR-U	Down-regulated (D)	FDR-D	Up- or down-regulated (E)	FDR-E
		Neighbors of radicicol	0.011	Neighbors of PIF3 (-)	0.034
		Neighbors of RPL	0.011	Neighbors of NRT2.1**	0.036
		Expression targets of RDR6	0.011	Regulators of sterol biosynthesis (-)	0.037
		Neighbors of heat shock protein	0.011	Targets of MIR393B (-)	0.039
		Regulators of cell growth	0.012	Neighbors of dibenziodolium (-)	0.040
		Neighbors of MIR166A	0.012	Neighbors of MIR156F (-)	0.040
		Neighbors of CUC2	0.012	Expression targets of WRKY (+)	0.040
		Binding partners of BES1	0.012	Expression targets of MKK3**	0.040
		Neighbors of BOP2	0.012	Expression targets of WRKY70**	0.040
		Regulators of gene silencing	0.012	Regulators of response to stress (-)	0.040
		Regulators of primordium initiation	0.012	Neighbors of CMT3 (-)	0.040
		Neighbors of CDK	0.013	Neighbors of dithiothreitol**	0.041
		Neighbors of PP2A	0.014	Neighbors of MIR393B (-)	0.042
		Binding partners of FIE	0.014	Neighbors of urea (-)	0.042
		Regulators of mitosis	0.014	Regulators of flavonol metabolism (-)	0.042
		Expression targets of HSF	0.014	Neighbors of D-GlcN**	0.044
		Regulators of leaf morphogenesis	0.015	Neighbors of FHY3 (-)	0.045
		Binding partners of GL3	0.015	Neighbors of PRL1 (-)	0.045
		Neighbors of latrunculin B	0.015	Neighbors of TT4**	0.046
		Neighbors of PRL1	0.015	Neighbors of ethylene treatment**	0.046
		Binding partners of CP47	0.016	Expression targets of HFR1 (-)	0.047
		Regulators of cell expansion	0.016	Neighbors of nectrolide (-)	0.047
		Regulators of preprophase band formation	0.017	Neighbors of LHY**	0.047

Table S5. Pathway Studio gene sets and subnetworks differentially expressed between paradormancy and endodormancy. 'Up-regulated' and 'down-regulated' gene sets are enriched for genes that either increased or decreased in gene expression, respectively. 'Up- or down-regulated' gene sets are enriched for genes that changed in gene expression, ignoring the direction of change of the individual genes. For the 'up- or down-regulated' gene sets, '**' denotes that the gene set was not significant in the other two analyses, whereas '+' and '-' indicate that the gene set was also enriched for genes that were up-regulated or down-regulated, respectively. Gene sets that were significant at an FDR q-value < 0.10 are identified by their Pathway Studio terms. FDR-U, FDR-D, and FDR-E are FDR q-values for up-regulated, down-regulated, and up- or down-regulated gene sets.

Up-regulated (U)	FDR-U	Down-regulated (D)	FDR-D	Up- or down-regulated (E)	FDR-E
		Binding partners of CDC2	0.017	Regulators of leaf patterning (-)	0.048
		Regulators of response to stress	0.017	Neighbors of thiamine**	0.049
		Binding partners of AGL24	0.017	Neighbors of EDR1**	0.050
		Neighbors of FHY3	0.019	Binding partners of DDB1A**	0.050
		Regulators of G1 phase	0.019	Neighbors of HFR1 (-)	0.051
		Regulators of floral organ development	0.019	Binding partners of SOS3**	0.052
		Regulators of trichome branching	0.019	Neighbors of PP2A (-)	0.052
		Regulators of ripening	0.019	Binding partners of E2FC**	0.053
		Neighbors of lammer kinase	0.020	Binding partners of BAK1**	0.053
		Expression targets of LUG	0.020	Neighbors of roscovitine (-)	0.054
		Neighbors of L-glutamine	0.020	Expression targets of calmodulin (-)	0.054
		Binding partners of GL1	0.020	Regulators of starch metabolism**	0.054
		Expression targets of STM	0.020	Regulators of pollination**	0.054
		Neighbors of DCL1	0.021	Neighbors of APRR9 (-)	0.054
		Binding partners of RPL	0.021	Regulators of floral organ development (-)	0.054
		Neighbors of MIR156C	0.021	Regulators of meristem growth (-)	0.056
		Targets of MIR156C	0.021	Binding partners of EGL3 (-)	0.057
		Expression targets of HFR1	0.021	Regulators of fatty acid elongation (-)	0.058
		Regulators of chromosome segregation	0.021	Neighbors of NPR3 (+)	0.058
		Expression targets of CUC2	0.022	Neighbors of macrofusine (-)	0.058
		Regulators of leaf shape	0.022	Expression targets of APRR9 (-)	0.058
		Targets of MIR156E	0.022	Neighbors of WRKY33 (+)	0.058
		Regulators of sterol biosynthesis	0.022	Neighbors of olomoucine (-)	0.060

Table S5. Pathway Studio gene sets and subnetworks differentially expressed between paradormancy and endodormancy. 'Up-regulated' and 'down-regulated' gene sets are enriched for genes that either increased or decreased in gene expression, respectively. 'Up- or down-regulated' gene sets are enriched for genes that changed in gene expression, ignoring the direction of change of the individual genes. For the 'up- or down-regulated' gene sets, '**' denotes that the gene set was not significant in the other two analyses, whereas '+' and '-' indicate that the gene set was also enriched for genes that were up-regulated or down-regulated, respectively. Gene sets that were significant at an FDR q-value < 0.10 are identified by their Pathway Studio terms. FDR-U, FDR-D, and FDR-E are FDR q-values for up-regulated, down-regulated, and up- or down-regulated gene sets.

Up-regulated (U)	FDR-U	Down-regulated (D)	FDR-D	Up- or down-regulated (E)	FDR-E
		Binding partners of AP1	0.022	Neighbors of leucine zipper**	0.060
		Neighbors of BLH4	0.022	Neighbors of CAT2**	0.060
		Neighbors of photosystem II reaction center	0.022	Neighbors of RCD1**	0.060
		Expression targets of CUC1	0.022	Neighbors of fenpropimorph (-)	0.061
		Regulators of pollen germination	0.022	Expression targets of GBF3 (-)	0.063
		Expression targets of HY5	0.023	Neighbors of cisplatin (-)	0.064
		Neighbors of chloramphenicol	0.023	Neighbors of DCL2**	0.066
		Neighbors of MIR156F	0.023	Neighbors of calcium channel**	0.066
		Regulators of secondary metabolism	0.023	Targets of MIR156H**	0.070
		Neighbors of CPC	0.023	Regulators of photobleaching**	0.071
		Binding partners of SMZ	0.023	Expression targets of CRY1**	0.071
		Neighbors of 14-3-3	0.023	Neighbors of STZ (-)	0.071
		Targets of MIR156D	0.023	Binding partners of PSBC (-)	0.072
		Neighbors of MIR156G	0.023	Neighbors of L-serine (-)	0.072
		Targets of MIR156F	0.024	Neighbors of GBF3 (-)	0.073
		Neighbors of HFR1	0.024	Regulators of histone methylation**	0.074
		Neighbors of MIR156E	0.024	Neighbors of lignin (-)	0.074
		Regulators of cell development	0.024	Neighbors of ACD6 (+)	0.074
		Regulators of microgametogenesis	0.024	Expression targets of STZ (-)	0.074
		Targets of MIR156G	0.024	Binding partners of HSP70 (-)	0.074
		Regulators of cell fate	0.024	Binding partners of FIE (-)	0.074
		Regulators of DNA recombination	0.025	Neighbors of aquaffin**	0.074
		Regulators of stomata conductance	0.026	Binding partners of JAZ10**	0.074

Table S5. Pathway Studio gene sets and subnetworks differentially expressed between paradormancy and endodormancy. 'Up-regulated' and 'down-regulated' gene sets are enriched for genes that either increased or decreased in gene expression, respectively. 'Up- or down-regulated' gene sets are enriched for genes that changed in gene expression, ignoring the direction of change of the individual genes. For the 'up- or down-regulated' gene sets, '**' denotes that the gene set was not significant in the other two analyses, whereas '+' and '-' indicate that the gene set was also enriched for genes that were up-regulated or down-regulated, respectively. Gene sets that were significant at an FDR q-value < 0.10 are identified by their Pathway Studio terms. FDR-U, FDR-D, and FDR-E are FDR q-values for up-regulated, down-regulated, and up- or down-regulated gene sets.

Up-regulated (U)	FDR-U	Down-regulated (D)	FDR-D	Up- or down-regulated (E)	FDR-E
		Targets of MIR393B	0.026	Expression targets of ACD6 (+)	0.075
		Regulators of pigmentation	0.026	Neighbors of antibiotic K 252A**	0.075
		Neighbors of CUC1	0.026	Neighbors of 14-3-3 (-)	0.076
		Targets of MIR156B	0.027	Binding partners of KAPP**	0.076
		Neighbors of MIR390A	0.028	Neighbors of RPL (-)	0.076
		Neighbors of H+	0.028	Neighbors of MIR156H**	0.077
		Binding partners of U1-70K	0.029	Regulators of vascularization (-)	0.078
		Neighbors of MIR156D	0.029	Expression targets of PR1**	0.078
		Neighbors of MIR393B	0.029	Neighbors of IM**	0.078
		Neighbors of MIR156B	0.029	Regulators of cell development (-)	0.079
		Neighbors of nectrolide	0.029	Neighbors of MIR157C**	0.081
		Protein modification targets of PP2A	0.029	Expression targets of AS2 (-)	0.081
		Neighbors of CDKN	0.029	Regulators of vacuolar transport**	0.082
		Regulators of cytosine methylation	0.029	Neighbors of FUS3**	0.082
		Binding partners of HSC70-1	0.030	Neighbors of AXR3**	0.082
		Regulators of leaf development	0.030	Neighbors of MIR157B**	0.082
		Regulators of vascularization	0.032	Neighbors of chloroplast GLTB**	0.082
		Neighbors of beta-aminobutyric acid	0.032	Neighbors of pi (lowercase)**	0.082
		Regulators of histogenesis	0.032	Neighbors of RGS1 (-)	0.082
		Regulators of leaf differentiation	0.032	Neighbors of MYB-factors (-)	0.082
		Neighbors of PIF3	0.032	Expression targets of MYB29 (-)	0.082
		Regulators of regulation of organ size	0.033	Neighbors of MIR157A**	0.082
		Regulators of DNA methylation	0.033	Neighbors of PKL**	0.082

Table S5. Pathway Studio gene sets and subnetworks differentially expressed between paradormancy and endodormancy. 'Up-regulated' and 'down-regulated' gene sets are enriched for genes that either increased or decreased in gene expression, respectively. 'Up- or down-regulated' gene sets are enriched for genes that changed in gene expression, ignoring the direction of change of the individual genes. For the 'up- or down-regulated' gene sets, '**' denotes that the gene set was not significant in the other two analyses, whereas '+' and '-' indicate that the gene set was also enriched for genes that were up-regulated or down-regulated, respectively. Gene sets that were significant at an FDR q-value < 0.10 are identified by their Pathway Studio terms. FDR-U, FDR-D, and FDR-E are FDR q-values for up-regulated, down-regulated, and up- or down-regulated gene sets.

Up-regulated (U)	FDR-U	Down-regulated (D)	FDR-D	Up- or down-regulated (E)	FDR-E
		Neighbors of MIR165A	0.035	Neighbors of WRKY70**	0.082
		Neighbors of olomoucine	0.036	Targets of MIR157B**	0.082
		Neighbors of MYB29	0.038	Expression targets of WRI1 (-)	0.083
		Neighbors of MAP3K	0.039	Regulators of RNA metabolism**	0.083
		Neighbors of PHB	0.039	Targets of MIR157C**	0.083
		Expression targets of APRR9	0.039	Neighbors of dicamba**	0.083
		Neighbors of PID	0.039	Neighbors of catalase**	0.084
		Neighbors of MIR408	0.039	Neighbors of MPK4**	0.084
		Neighbors of MIR157D	0.039	Regulators of organ development (-)	0.084
		Neighbors of RGD3	0.040	Neighbors of cyclic nucleotides**	0.084
		Neighbors of COI1	0.041	Regulators of trichome density (-)	0.084
		Expression targets of MYB29	0.042	Neighbors of PHV (-)	0.084
		Neighbors of APRR9	0.042	Neighbors of lanthanum (-)	0.084
		Neighbors of CYCD3;1	0.043	Neighbors of PDF1.2**	0.085
		Regulators of DNA replication	0.043	Neighbors of PIF4 (-)	0.085
		Targets of MIR157D	0.043	Neighbors of MYB29 (-)	0.085
		Neighbors of phosphatidylglycerol	0.044	Regulators of chromosome segregation (-)	0.085
		Regulators of response to auxin stimulus	0.044	Neighbors of JAR1 (-)	0.085
		Neighbors of UV	0.047	Neighbors of GL1**	0.085
		Neighbors of brassinosteroids	0.048	Neighbors of CDC2 (-)	0.086
		Binding partners of CBL2	0.049	Targets of MIR157A**	0.086
		Expression targets of TTG1	0.050	Neighbors of H+ (-)	0.086
		Regulators of internode growth	0.050	Regulators of seed size (-)	0.086

Table S5. Pathway Studio gene sets and subnetworks differentially expressed between paradormancy and endodormancy. 'Up-regulated' and 'down-regulated' gene sets are enriched for genes that either increased or decreased in gene expression, respectively. 'Up- or down-regulated' gene sets are enriched for genes that changed in gene expression, ignoring the direction of change of the individual genes. For the 'up- or down-regulated' gene sets, '**' denotes that the gene set was not significant in the other two analyses, whereas '+' and '-' indicate that the gene set was also enriched for genes that were up-regulated or down-regulated, respectively. Gene sets that were significant at an FDR q-value < 0.10 are identified by their Pathway Studio terms. FDR-U, FDR-D, and FDR-E are FDR q-values for up-regulated, down-regulated, and up- or down-regulated gene sets.

Up-regulated (U)	FDR-U	Down-regulated (D)	FDR-D	Up- or down-regulated (E)	FDR-E
		Binding partners of PHYA	0.052	Neighbors of KAPP**	0.088
		Neighbors of roscovitine	0.052	Neighbors of GPRI1**	0.088
		Neighbors of MEJA	0.052	Neighbors of SOS3**	0.092
		Binding partners of AGL20	0.052	Neighbors of ATHK1**	0.092
		Neighbors of AHK3	0.052	Expression targets of leucine zipper**	0.096
		Neighbors of FT	0.053	Expression targets of COI1 (-)	0.097
		Neighbors of floraltone	0.054	Neighbors of ERF1A**	0.097
		Regulators of shoot development	0.054	Neighbors of geldanamycin (-)	0.098
		Neighbors of HDA6	0.056	Neighbors of laminaran (+)	0.099
		Expression targets of AS1	0.056	Regulators of transpiration (-)	0.099
		Expression targets of RPL	0.056		
		Neighbors of cisplatin	0.057		
		Neighbors of ATR	0.057		
		Neighbors of lignin	0.058		
		Binding partners of HSP81-2	0.058		
		Regulators of seed size	0.058		
		Neighbors of fenpropimorph	0.060		
		Regulators of pigment accumulation	0.061		
		Neighbors of ARGOS	0.062		
		Expression targets of WRI1	0.062		
		Neighbors of cytokinin	0.062		
		Neighbors of ABF2	0.063		
		Regulators of organ development	0.064		

Table S5. Pathway Studio gene sets and subnetworks differentially expressed between paradormancy and endodormancy. 'Up-regulated' and 'down-regulated' gene sets are enriched for genes that either increased or decreased in gene expression, respectively. 'Up- or down-regulated' gene sets are enriched for genes that changed in gene expression, ignoring the direction of change of the individual genes. For the 'up- or down-regulated' gene sets, '**' denotes that the gene set was not significant in the other two analyses, whereas '+' and '-' indicate that the gene set was also enriched for genes that were up-regulated or down-regulated, respectively. Gene sets that were significant at an FDR q-value < 0.10 are identified by their Pathway Studio terms. FDR-U, FDR-D, and FDR-E are FDR q-values for up-regulated, down-regulated, and up- or down-regulated gene sets.

Up-regulated (U)	FDR-U	Down-regulated (D)	FDR-D	Up- or down-regulated (E)	FDR-E
		Neighbors of sucrose	0.064		
		Neighbors of histone	0.064		
		Regulators of maintenance of DNA methylation	0.064		
		Neighbors of STZ	0.065		
		Regulators of meristem initiation	0.065		
		Neighbors of RGS1	0.065		
		Neighbors of grelutin	0.065		
		Regulators of primordium development	0.066		
		Regulators of trichome morphogenesis	0.066		
		Binding partners of TIR1	0.066		
		Regulators of meristem maintenance	0.066		
		Neighbors of H+-transporting two-sector atpase	0.066		
		Regulators of meristem size	0.067		
		Neighbors of lanthanum	0.067		
		Neighbors of CDC2	0.067		
		Neighbors of PHV	0.067		
		Expression targets of DET1	0.067		
		Neighbors of ARF2	0.067		
		Neighbors of MIR166G	0.067		
		Neighbors of calmodulin	0.067		
		Neighbors of ER	0.068		
		Neighbors of S-adenosylmethionine	0.068		
		Regulators of cytoskeleton organization and biogenesis	0.068		

Table S5. Pathway Studio gene sets and subnetworks differentially expressed between paradormancy and endodormancy. 'Up-regulated' and 'down-regulated' gene sets are enriched for genes that either increased or decreased in gene expression, respectively. 'Up- or down-regulated' gene sets are enriched for genes that changed in gene expression, ignoring the direction of change of the individual genes. For the 'up- or down-regulated' gene sets, '**' denotes that the gene set was not significant in the other two analyses, whereas '+' and '-' indicate that the gene set was also enriched for genes that were up-regulated or down-regulated, respectively. Gene sets that were significant at an FDR q-value < 0.10 are identified by their Pathway Studio terms. FDR-U, FDR-D, and FDR-E are FDR q-values for up-regulated, down-regulated, and up- or down-regulated gene sets.

Up-regulated (U)	FDR-U	Down-regulated (D)	FDR-D	Up- or down-regulated (E)	FDR-E
		Neighbors of ROP1	0.068		
		Neighbors of glutathione	0.068		
		Neighbors of JA	0.068		
		Targets of MIR165A	0.068		
		Regulators of regulation of cell size	0.068		
		Neighbors of KAN	0.069		
		Neighbors of PIF4	0.069		
		Neighbors of JAR1	0.069		
		Targets of MIR165B	0.069		
		Regulators of root growth	0.069		
		Neighbors of MEK2	0.070		
		Neighbors of AFC2	0.070		
		Binding partners of CKS1	0.070		
		Binding partners of ARF1	0.074		
		Neighbors of mastoparan	0.075		
		Regulators of cell proliferation	0.075		
		Neighbors of paraquat	0.075		
		Neighbors of TT8	0.075		
		Neighbors of MIR165B	0.075		
		Regulators of translation	0.075		
		Neighbors of dibenziodolium	0.076		
		Expression targets of STZ	0.077		
		Neighbors of ETT	0.077		

Table S5. Pathway Studio gene sets and subnetworks differentially expressed between paradormancy and endodormancy. 'Up-regulated' and 'down-regulated' gene sets are enriched for genes that either increased or decreased in gene expression, respectively. 'Up- or down-regulated' gene sets are enriched for genes that changed in gene expression, ignoring the direction of change of the individual genes. For the 'up- or down-regulated' gene sets, '**' denotes that the gene set was not significant in the other two analyses, whereas '+' and '-' indicate that the gene set was also enriched for genes that were up-regulated or down-regulated, respectively. Gene sets that were significant at an FDR q-value < 0.10 are identified by their Pathway Studio terms. FDR-U, FDR-D, and FDR-E are FDR q-values for up-regulated, down-regulated, and up- or down-regulated gene sets.

Up-regulated (U)	FDR-U	Down-regulated (D)	FDR-D	Up- or down-regulated (E)	FDR-E
		Binding partners of HSP70	0.077		
		Neighbors of AHP1	0.077		
		Neighbors of G-protein	0.078		
		Regulators of plant infection	0.078		
		Regulators of lipid transport	0.078		
		Regulators of root differentiation	0.078		
		Neighbors of NADPH	0.079		
		Regulators of seed abscission	0.080		
		Neighbors of basic-helix-loop-helix protein	0.080		
		Neighbors of MIR166F	0.080		
		Neighbors of MIR160A	0.080		
		Targets of MIR166C	0.080		
		Binding partners of PIN1	0.080		
		Targets of MIR166F	0.081		
		Targets of MIR166E	0.081		
		Targets of MIR166A	0.081		
		Regulators of morphogenesis	0.081		
		Targets of MIR166D	0.082		
		Neighbors of urea	0.082		
		Protein modification targets of PRMT11	0.082		
		Regulators of transpiration	0.082		
		Regulators of nodulation	0.082		
		Regulators of chromatin remodeling	0.082		

Table S5. Pathway Studio gene sets and subnetworks differentially expressed between paradormancy and endodormancy. 'Up-regulated' and 'down-regulated' gene sets are enriched for genes that either increased or decreased in gene expression, respectively. 'Up- or down-regulated' gene sets are enriched for genes that changed in gene expression, ignoring the direction of change of the individual genes. For the 'up- or down-regulated' gene sets, '**' denotes that the gene set was not significant in the other two analyses, whereas '+' and '-' indicate that the gene set was also enriched for genes that were up-regulated or down-regulated, respectively. Gene sets that were significant at an FDR q-value < 0.10 are identified by their Pathway Studio terms. FDR-U, FDR-D, and FDR-E are FDR q-values for up-regulated, down-regulated, and up- or down-regulated gene sets.

**Up-regulated (U)**	**FDR-U**	**Down-regulated (D)**	**FDR-D**	**Up- or down-regulated (E)**	**FDR-E**
		Neighbors of MIR396A	0.082		
		Regulators of kinetochore assembly	0.082		
		Targets of MIR166B	0.082		
		Expression targets of COI1	0.082		
		Regulators of exine formation	0.083		
		Targets of MIR166G	0.083		
		Neighbors of deacetylchitin	0.083		
		Neighbors of macrofusine	0.084		
		Neighbors of MIR166E	0.085		
		Neighbors of auxins	0.085		
		Regulators of stomatal complex formation	0.085		
		Binding partners of PSBC	0.086		
		Regulators of mRNA degradation	0.086		
		Neighbors of MYB28	0.086		
		Neighbors of MIR166D	0.086		
		Neighbors of MIR166B	0.087		
		Binding partners of SEP3	0.088		
		Expression targets of BLH8	0.088		
		Regulators of trichome density	0.088		
		Neighbors of paclobutrazol	0.089		
		Neighbors of STM	0.090		
		Neighbors of HPT1	0.090		
		Neighbors of PRMT11	0.090		

Table S5. Pathway Studio gene sets and subnetworks differentially expressed between paradormancy and endodormancy. 'Up-regulated' and 'down-regulated' gene sets are enriched for genes that either increased or decreased in gene expression, respectively. 'Up- or down-regulated' gene sets are enriched for genes that changed in gene expression, ignoring the direction of change of the individual genes. For the 'up- or down-regulated' gene sets, '**' denotes that the gene set was not significant in the other two analyses, whereas '+' and '-' indicate that the gene set was also enriched for genes that were up-regulated or down-regulated, respectively. Gene sets that were significant at an FDR q-value < 0.10 are identified by their Pathway Studio terms. FDR-U, FDR-D, and FDR-E are FDR q-values for up-regulated, down-regulated, and up- or down-regulated gene sets.

Up-regulated (U)	FDR-U	Down-regulated (D)	FDR-D	Up- or down-regulated (E)	FDR-E
		Neighbors of MIR166C	0.091		
		Neighbors of brassinolide treatment	0.091		
		Neighbors of magnogene	0.092		
		Binding partners of BLH8	0.095		
		Neighbors of HYH	0.096		
		Neighbors of CKX3	0.096		
		Targets of MIR408	0.097		
		Regulators of trichome patterning	0.097		
		Neighbors of AGO7	0.097		
		Regulators of mating	0.097		
		Neighbors of BLH8	0.099		

Table S6. Pathway Studio gene sets and subnetworks differentially expressed between endodormancy and ecodormancy. 'Up-regulated' and 'down-regulated' gene sets are enriched for genes that either increased or decreased in gene expression, respectively. 'Up- or down-regulated' gene sets are enriched for genes that changed in gene expression, ignoring the direction of change of the individual genes. For the 'up- or down-regulated' gene sets, '**' denotes that the gene set was not significant in the other two analyses, whereas '+' and '-' indicate that the gene set was also enriched for genes that were up-regulated or down-regulated, respectively. Gene sets that were significant at an FDR q-value < 0.10 are identified by their Pathway Studio terms. FDR-U, FDR-D, and FDR-E are FDR q-values for up-regulated, down-regulated, and up- or down-regulated gene sets.

**Up-regulated (U)**	**FDR-U**	**Down-regulated (D)**	**FDR-D**	**Up- or down-regulated (E)**	**FDR-E**
Neighbors of MIR396A	0.008	Neighbors of MIR169N	0.001	Targets of MIR169L (-)	0.003
Neighbors of ARF2	0.010	Neighbors of MIR169M	0.001	Neighbors of MIR169H (-)	0.003
Regulators of histogenesis	0.013	Targets of MIR169N	0.001	Targets of MIR169K (-)	0.003
Neighbors of SEU	0.020	Targets of MIR169B	0.001	Targets of MIR169B (-)	0.003
Expression targets of ARF2	0.021	Targets of MIR169H	0.001	Targets of MIR169I (-)	0.003
Regulators of primordium initiation	0.023	Targets of MIR169K	0.001	Neighbors of MIR169L (-)	0.003
Neighbors of ARGOS	0.023	Neighbors of EIN3	0.001	Neighbors of MIR169B (-)	0.003
Neighbors of RPL	0.027	Neighbors of MIR169I	0.001	Neighbors of MIR169I (-)	0.003
Regulators of cytosine methylation	0.039	Neighbors of MIR169B	0.001	Neighbors of MIR169N (-)	0.003
Targets of MIR396A	0.041	Neighbors of XRN4	0.001	Neighbors of MIR169A (-)	0.003
Expression targets of ANT	0.041	Targets of MIR169M	0.001	Targets of MIR169H (-)	0.003
Neighbors of steroids	0.057	Neighbors of MIR169A	0.001	Targets of MIR169A (-)	0.004
Neighbors of latrunculin B	0.057	Neighbors of MIR169K	0.001	Targets of MIR169C (-)	0.004
Neighbors of L-serine	0.058	Targets of MIR169C	0.001	Targets of MIR169M (-)	0.004
Neighbors of ROP1	0.059	Neighbors of MIR169L	0.001	Neighbors of MIR169C (-)	0.004
Regulators of stomata conductance	0.061	Targets of MIR169L	0.001	Targets of MIR169N (-)	0.004
Expression targets of SEU	0.061	Neighbors of casein kinase II	0.001	Neighbors of MIR169M (-)	0.005
Regulators of cell fate	0.062	Neighbors of MIR169C	0.001	Targets of MIR169J (-)	0.006
Regulators of meristem organization	0.062	Protein modification targets of casein kinase II	0.002	Neighbors of MIR169K (-)	0.006
Regulators of organ development	0.063	Targets of MIR169A	0.002	Regulators of histogenesis (+)	0.007
Regulators of internode growth	0.063	Neighbors of ABF3	0.002	Neighbors of MIR169J (-)	0.008
Neighbors of BOP1	0.063	Neighbors of EIL1	0.002	Neighbors of ARF2 (+)	0.009
Expression targets of E2F	0.064	Targets of MIR169I	0.002	Binding partners of BAK1**	0.011

Table S6. Pathway Studio gene sets and subnetworks differentially expressed between endodormancy and ecodormancy. 'Up-regulated' and 'down-regulated' gene sets are enriched for genes that either increased or decreased in gene expression, respectively. 'Up- or down-regulated' gene sets are enriched for genes that changed in gene expression, ignoring the direction of change of the individual genes. For the 'up- or down-regulated' gene sets, '**' denotes that the gene set was not significant in the other two analyses, whereas '+' and '-' indicate that the gene set was also enriched for genes that were up-regulated or down-regulated, respectively. Gene sets that were significant at an FDR q-value < 0.10 are identified by their Pathway Studio terms. FDR-U, FDR-D, and FDR-E are FDR q-values for up-regulated, down-regulated, and up- or down-regulated gene sets.

**Up-regulated (U)**	**FDR-U**	**Down-regulated (D)**	**FDR-D**	**Up- or down-regulated (E)**	**FDR-E**
Neighbors of JLO	0.066	Neighbors of SOS3	0.002	Neighbors of AMP1 (+)	0.013
Regulators of organ formation	0.069	Neighbors of MIR169H	0.002	Neighbors of calcium channel**	0.015
Neighbors of AMP1	0.070	Neighbors of MIR169J	0.002	Expression targets of calcium channel**	0.016
Neighbors of basic-helix-loop-helix protein	0.070	Neighbors of PHOT2	0.002	Neighbors of MIR396A (+)	0.018
Regulators of sugar transport	0.071	Targets of MIR169J	0.004	Neighbors of ANP1**	0.018
Neighbors of KAPP	0.072	Regulators of autophagy	0.007	Neighbors of ARGOS (+)	0.020
Neighbors of RHO	0.074	Neighbors of glutathione transferase	0.009	Neighbors of gamma-thio-ATP**	0.025
Binding partners of LUG	0.076	Neighbors of LHY	0.009	Neighbors of starch**	0.038
Neighbors of grelutin	0.087	Neighbors of EIN2	0.010	Neighbors of L-serine (+)	0.038
Neighbors of RDR6	0.088	Regulators of photoperiodism	0.010	Expression targets of HOMEO**	0.045
Regulators of cuticle development	0.088	Neighbors of spermine	0.011	Neighbors of MKK4**	0.046
Regulators of integument development	0.092	Protein modification targets of glutathione transferase	0.012	Targets of MIR396A (+)	0.078
Regulators of protoxylem development	0.092	Neighbors of calyculin A	0.012	Neighbors of IAA1**	0.084
Regulators of regulation of cell shape	0.092	Neighbors of HD1	0.012		
Neighbors of BOP2	0.093	Expression targets of EIN3	0.013		
Neighbors of ANT	0.094	Neighbors of EREBP	0.013		
Regulators of flower identity	0.095	Neighbors of SOS1	0.018		
Binding partners of PSAK	0.095	Binding partners of ARA6	0.019		
Neighbors of polycomb complex	0.096	Neighbors of FUS9	0.021		
Regulators of maintenance of DNA methylation	0.099	Neighbors of SOS2	0.022		
		Neighbors of antibiotic K 252A	0.022		
		Neighbors of blue light photoreceptor	0.031		
		Neighbors of ethylene treatment	0.037		

Table S6. Pathway Studio gene sets and subnetworks differentially expressed between endodormancy and ecodormancy. 'Up-regulated' and 'down-regulated' gene sets are enriched for genes that either increased or decreased in gene expression, respectively. 'Up- or down-regulated' gene sets are enriched for genes that changed in gene expression, ignoring the direction of change of the individual genes. For the 'up- or down-regulated' gene sets, '**' denotes that the gene set was not significant in the other two analyses, whereas '+' and '-' indicate that the gene set was also enriched for genes that were up-regulated or down-regulated, respectively. Gene sets that were significant at an FDR q-value < 0.10 are identified by their Pathway Studio terms. FDR-U, FDR-D, and FDR-E are FDR q-values for up-regulated, down-regulated, and up- or down-regulated gene sets.

Up-regulated (U)	FDR-U	Down-regulated (D)	FDR-D	Up- or down-regulated (E)	FDR-E
		Binding partners of OBF5	0.039		
		Neighbors of EDS16	0.040		
		Regulators of plant stress	0.041		
		Binding partners of ARA7	0.043		
		Neighbors of antimycin A	0.060		
		Neighbors of ethylene receptor	0.061		
		Expression targets of EIN2	0.062		
		Neighbors of LWD2	0.063		
		Regulators of ER unfolded protein response	0.064		
		Neighbors of tunicamycin	0.067		
		Expression targets of HXK1	0.067		
		Expression targets of WRKY	0.067		
		Neighbors of salicylate	0.068		
		Neighbors of RHL41	0.068		
		Regulators of photomorphogenesis	0.068		
		Neighbors of NPR1	0.068		
		Neighbors of ZTL	0.072		
		Expression targets of ZTL	0.072		
		Neighbors of ANAC019	0.073		
		Regulators of cryptochrome response	0.073		
		Expression targets of ABI2	0.073		
		Neighbors of MEK1	0.075		
		Neighbors of leucine zipper	0.076		

Table S6. Pathway Studio gene sets and subnetworks differentially expressed between endodormancy and ecodormancy. 'Up-regulated' and 'down-regulated' gene sets are enriched for genes that either increased or decreased in gene expression, respectively. 'Up- or down-regulated' gene sets are enriched for genes that changed in gene expression, ignoring the direction of change of the individual genes. For the 'up- or down-regulated' gene sets, '**' denotes that the gene set was not significant in the other two analyses, whereas '+' and '-' indicate that the gene set was also enriched for genes that were up-regulated or down-regulated, respectively. Gene sets that were significant at an FDR q-value < 0.10 are identified by their Pathway Studio terms. FDR-U, FDR-D, and FDR-E are FDR q-values for up-regulated, down-regulated, and up- or down-regulated gene sets.

**Up-regulated (U)**	**FDR-U**	**Down-regulated (D)**	**FDR-D**	**Up- or down-regulated (E)**	**FDR-E**
		Binding partners of CUL1	0.076		
		Neighbors of MYB58	0.077		
		Binding partners of TGA6	0.077		
		Neighbors of ATAN11	0.077		
		Neighbors of EIN4	0.077		
		Neighbors of NADH	0.077		
		Regulators of salinty response	0.077		
		Binding partners of OEP80	0.081		
		Expression targets of ANAC019	0.082		
		Regulators of skotomorphogenesis	0.087		
		Neighbors of MEK2	0.089		
		Neighbors of MPK3	0.089		
		Binding partners of FKF1	0.093		
		Expression targets of CNX5	0.094		
		Neighbors of ZLLLAL	0.100		

Table S7. Hormone gene sets differentially expressed between paradormancy and endodormancy. 'Up-regulated' and 'down-regulated' gene sets are enriched for genes that either increased or decreased in gene expression, respectively. 'Up- or down-regulated' gene sets are enriched for genes that changed in gene expression, ignoring the direction of change of the individual genes. For the 'up- or down-regulated' gene sets, '**' denotes that the gene set was not significant in the other two analyses, whereas '+' and '-' indicate that the gene set was also enriched for genes that were up-regulated or down-regulated, respectively. Gene sets that were significant at an FDR q-value < 0.10 are identified by their corresponding phytohormone. FDR-U, FDR-D, and FDR-E are FDR q-values for up-regulated, down-regulated, and up- or down-regulated gene sets.

Up-regulated (U)	FDR-U	Down-regulated (D)	FDR-D	Up- or down-regulated (E)	FDR-E
Gibberellin	0.016	Auxin	0.000	Salicylic acid (-)	0.000
Ethylene	0.028	Brassinosteroid	0.034	Auxin (-)	0.055
		Salicylic acid	0.043		

Table S8. Hormone gene sets differentially expressed between endodormancy and ecodormancy. 'Up-regulated' and 'down-regulated' gene sets are enriched for genes that either increased or decreased in gene expression, respectively. 'Up- or down-regulated' gene sets are enriched for genes that changed in gene expression, ignoring the direction of change of the individual genes. For the 'up- or down-regulated' gene sets, '**' denotes that the gene set was not significant in the other two analyses, whereas '+' and '-' indicate that the gene set was also enriched for genes that were up-regulated or down-regulated, respectively. Gene sets that were significant at an FDR q-value < 0.10 are identified by their corresponding phytohormone. FDR-U, FDR-D, and FDR-E are FDR q-values for up-regulated, down-regulated, and up- or down-regulated gene sets.

Up-regulated (U)	FDR-U	Down-regulated (D)	FDR-D	Up- or down-regulated (E)	FDR-E
Auxin	0.039	Ethylene	0.000		
